# Long‐term monitoring and remote sensing reveal waterbird colony collapses with rising lake levels and multiple stressors

**DOI:** 10.1002/eap.70314

**Published:** 2026-09-28

**Authors:** Jennifer L. Fuller, Erin Ford, Ava Landgraf, Johannes Foufopoulos, Andrew Gronewold, Karen M. Alofs

**Affiliations:** ^1^ School for Environment and Sustainability, The University of Michigan Ann Arbor Michigan USA; ^2^ Audubon Great Lakes Chicago Illinois USA; ^3^ Detroit Bird Alliance Detroit Michigan USA; ^4^ Department of Civil and Environmental Engineering The University of Michigan Ann Arbor Michigan USA; ^5^ Department of Earth and Environmental Sciences The University of Michigan Ann Arbor Michigan USA; ^6^ Present address: Independent Researcher Wyandotte Michigan USA; ^7^ Present address: National Audubon Society New York New York USA

**Keywords:** black tern (*Chlidonias niger*), climate change, coastal squeeze, coastal wetlands, common reed (*Phragmites australis*), ecological resilience, Great Lakes, hatching success, invasive species, lake level fluctuations, multiple stressors, wetland conservation

## Abstract

Global climate change is expected to exacerbate existing environmental stressors and increasingly impact biodiversity. While likely widespread, changes in wildlife populations resulting from the interaction of climate change and other environmental stressors remain poorly understood. We investigated climate‐mediated lake level fluctuations in coastal wetland ecosystems undergoing shoreline development and species invasions in the Laurentian Great Lakes. We evaluated the potential of multiple stressors to reduce ecological resilience and generate alternative stable states, by examining effects on a declining wetland habitat indicator species, the Black Tern (*Chlidonias niger*). We monitored one of the largest breeding Black Tern colonies in the Great Lakes over 8 years (2013–2020), recording population size, nest location and hatch success, clutch initiation date, and nest habitat characteristics (relative initial nest water depth, mean NDVI and % dense vegetation within a 7 and 12‐m radius, and distance to open water and developed land). We applied a generalized linear mixed‐effects model (GLMM) to determine hatch success response to biological and habitat predictors. We mapped the GLMM's predictors to quantify the spatial extent of hatch success likelihood and used a second GLMM to relate sub‐colony breeding pair abundance to habitat variables derived from the spatial model. From 2013 to 2020, the mean breeding season lake level (May–July) increased by 1.03 m, coinciding with a 55% decline in hatch success rate and 77% decline in breeding pairs. Hatching success was negatively related to clutch initiation date, relative initial nest water depth, and % dense vegetation, and positively related to distance from the main lake and mean NDVI. The GLMM indicated breeding pair abundance was positively related to the area of sub‐colony habitat with >50% hatch success likelihood. Our findings demonstrate rapid hydrological change, coastline development, and common reed (*Phragmites australis*) invasion reduced habitat migration potential and available nesting habitat, likely contributing to near abandonment of the colony. While future hydrological projections are uncertain, we expect Black Tern colony populations to fluctuate in response to lake levels in the region. Our study exemplifies how multiple stressors can undermine wetland resilience to climate change and present a conservation challenge for vulnerable wetland taxa.

## INTRODUCTION

Recent estimates suggest that nearly 30% of species have become globally threatened or extinct over the last 500 years, resulting in worldwide losses in biodiversity and ecosystem functioning (Isbell et al., 2023). The greatest threats to global biodiversity are anthropogenic stressors, including habitat loss, invasive species, pollution, and climate change (Hogue & Breon, 2022; Orr et al., 2020). Most ecosystems face a suite of simultaneous threats (hereafter, “multiple stressors”). Understanding and addressing these often complex or unforeseen combined effects is critically important for effective conservation management (Orr et al., 2020; Pirotta et al., 2022). Stressors with additive and synergistic effects can deteriorate ecosystem resilience to climate perturbations and generate rapid regime shifts towards undesirable alternative states with reduced biodiversity (Beisner et al., 2003; Côté et al., 2016; Folke et al., 2004). To reduce, reverse, or prevent undesirable regime shifts, scientists and land managers must identify and correctly address offending stressors through the lens of ecosystem resilience (Côté & Darling, 2010), which is the ability for a system to absorb and adjust to climate‐driven disturbances while maintaining its core functions, structure, identity, and internal processes (Walker et al., 2004).

Wetlands are recognized for their high natural resilience, but human activities threaten their ability to adapt and recover from disturbances (Jacob et al., 2025). Understanding how multiple stressors affect wetland functions is therefore key to effective management and safeguarding a critical source of global carbon sequestration, ecosystem services, and biodiversity. Despite only comprising 7% of the earth's terrestrial landcover (Convention on Wetlands, 2021), over 30% of terrestrial soil carbon is sequestered by peatlands, marshes, swamps, and coastal wetlands globally (Mitsch, 2016; USGCRP, 2018). Both saline and freshwater wetlands provide essential ecosystem services such as storm protection, reduced shoreline erosion, and water purification (Barbier et al., 2011). Wetlands also support roughly 40% of earth's species during their life cycle (Convention on Wetlands, 2021). In the Laurentian Great Lakes, freshwater coastal wetlands are home to 75% of the lakes' fish species (Trebitz et al., 2019) and 350 species of migratory and breeding birds (Bielke et al., 2021). These wetlands also support one‐third of the Great Lakes primary productivity (Brazner et al., 2001) and provide water purification, carbon sequestration, and coastal storm protection to a coastal population of over 19 million people and a watershed population of over 38 million (Crowell et al., 2007; Fergen et al., 2022; Meli et al., 2014).

Despite their ecological importance, global wetland ecosystems have been increasingly lost or degraded (Davidson, 2018), with an estimated 35% decline in 1970 and 2015 worldwide (Gardner & Finlayson, 2018). Wetlands are also threatened by multiple stressors and are sensitive to climate change (Millennium Ecosystem Assessment, 2005). Great Lakes coastal wetlands face many of these threats, reducing their capacity to provide ecosystem services and wildlife habitat (Gao et al., 2023; Millennium Ecosystem Assessment, 2005). These threats include invasive species, human development, and pollutants (Danz et al., 2007; Millennium Ecosystem Assessment, 2005; Tulbure et al., 2007), contributing to a 50% loss of their historical range (Krieger, 1992). Climate change also threatens to severely alter coastal wetlands through extreme lake level variation. Warming atmospheric temperatures have increasingly altered the Great Lakes regional water budget, resulting in record‐breaking lake level fluctuations (Clites et al., 2014; Gronewold et al., 2016; Gronewold et al., 2021; VanDeWeghe et al., 2022).

Although water level fluctuations play a beneficial role in mediating zonation, sustaining plant biodiversity, and preventing vegetation oversaturation for coastal and inland wetlands (Bansal & Lishawa, 2019; Gerson, 1988; Wilcox, 2004; Figure 1), this relationship is often complicated by the presence of wetland stressors such as invasive species, development, and climate change (Danz et al., 2007). For example, invasive common reed (*Phragmites australis*) and cattail (*Typha x glauca, Typha angustifolia*) exhibit competitive dominance under extreme hydrological swings or stabilization (Bansal & Lishawa, 2019; Bourgeau‐Chavez et al., 2013; Tulbure & Johnston, 2010; Wilcox et al., 2008). Invasive species and coastal development can also create barriers to landward wetland migration during lake level rise (i.e., “coastal squeeze”; Pontee, 2013; Anderson et al., 2023; Tozer et al., 2024). To assess the impacts of these wetland stressors on ecosystem health and inform management strategies, researchers can monitor the presence, abundance, and productivity of wildlife species that are sensitive to changes in wetland quality, extent, disturbance, and hydrologic dynamics. These include avian species such as Black Tern (*Chlidonias niger*), Black‐crowned Night‐Heron (*Nycticorax nycticorax*), Least Bittern (*Ixobrychus exilis*), Marsh Wren (*Cistothorus palustris*), Pied‐billed Grebe (*Podilymbus podiceps*), and Sora (*Porzana carolina*), which are furthermore sensitive indicators as they require productive wetlands with high environmental heterogeneity in order to support their unique foraging and breeding strategies (Craig & Beal, 1992; Desgranges et al., 2006; Grand et al., 2020; Kantrud & Stewart, 1984; Saunders et al., 2019).

**FIGURE 1:**
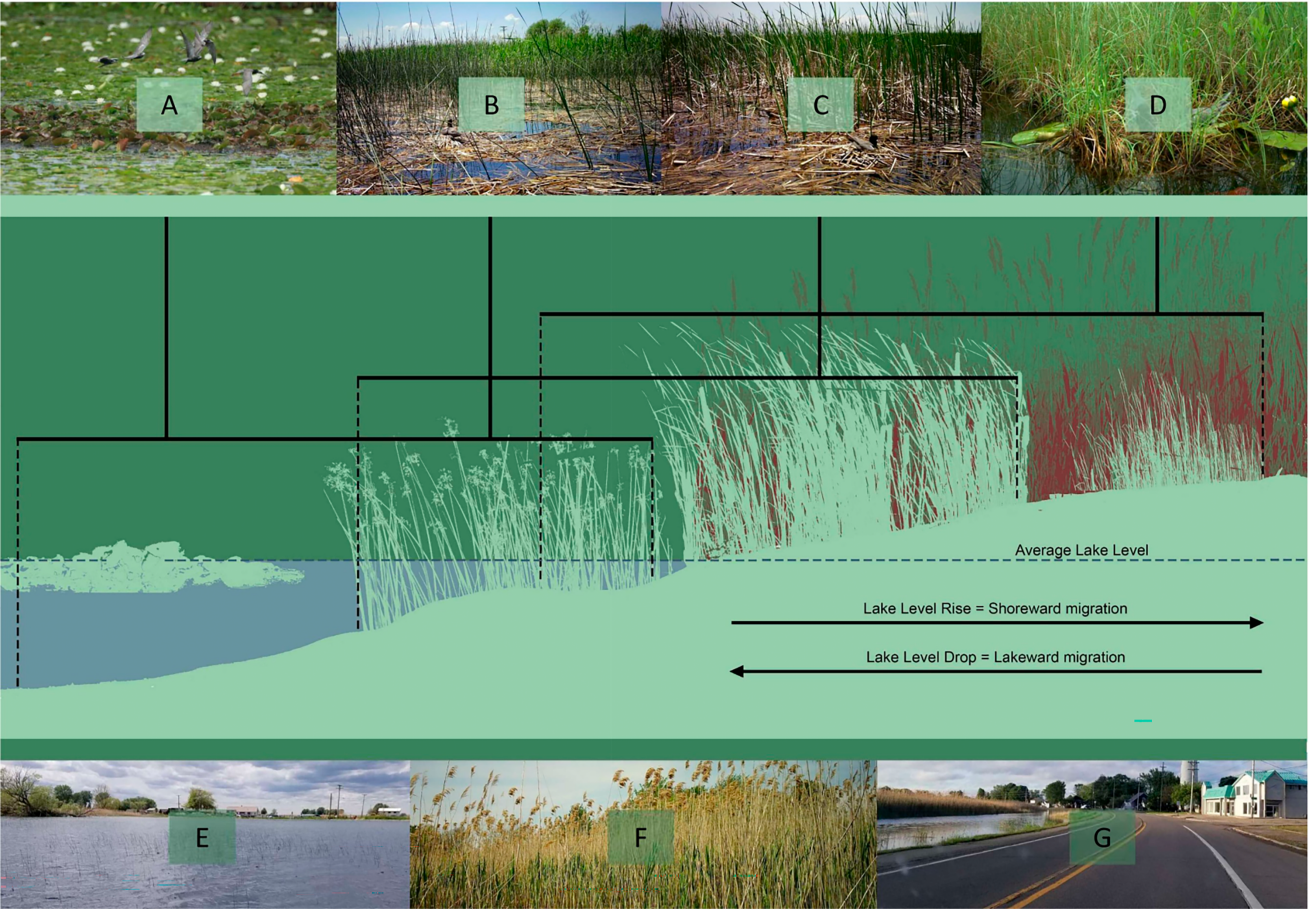
Graphic representation of Black Tern nesting habitat zones in order of increasing proximity to land: (A) water lily (*Nuphar* spp., *Nymphea* spp.), (B) bulrush (*Scirpus* spp., *Schoenoplectus* sp.), (C) cattail (*Typha* spp.), and (D) sedge (*Carex* spp.) dominant. As lake levels drop, plant communities migrate lakeward unless propagation is limited by high hydraulic energy, boat traffic, and/or deep water (E). During drought periods and migration, shallow and exposed soil that no longer supports natural vegetation can become invaded by common reed and cattail (F, main image: Red). As lake levels rise again, communities will move up the shoreline, but can be blocked by common reed establishments or shoreline developments (G). Adapted from (Bolsenga & Herdendorf, 1993; Mortsch et al., 2006). Credit for photos (A–G) and illustration (center): Jennifer L. Fuller.

Historically, Black Terns and several other Great Lakes marsh bird species have faced substantial population declines due to habitat loss and degradation (Mortsch et al., 2006; Robichaud & Rooney, 2017; Soulliere et al., 2018; Wyman & Cuthbert, 2017) with approximately six marsh‐breeding birds declining 2%–8% annually from 1995 to 2018 (Tozer, 2020). In North America, breeding Black Tern populations fell 2.4% annually between 1966 and 2019, and 7.6% in the state of Michigan (Sauer et al., 2019). Additionally, in the Great Lakes, 90% of historical Black Tern colonies were abandoned between 1991 and 2009 (Wyman & Cuthbert, 2017). However, these trends improved between 2011 and 2021, where nine marsh bird species, including Black Terns, increased 8%–37% annually across the Great Lakes Basin, potentially due to higher lake levels improving coastal wetland interspersion (Tozer et al., 2024). Because Black Terns spend much of their annual cycle migrating and wintering in Central and South America (Heath et al., 2020), threats likely extend beyond breeding grounds. An integrated population model from Lake St. Clair and Wigwam Bay, Michigan, identified adult survival as the strongest driver of population size and emphasized the importance of conservation along migratory routes and wintering areas. However, high interannual variability in demographic rates may limit population recovery; therefore, stabilizing and increasing fecundity through breeding habitat protection and restoration remains critical (Davis et al., 2023).

Few, if any, studies have examined how multiple stressors and climate change impact the breeding response of Black Terns and other colonial marsh birds. Wetland resilience to multiple stressors likely influences habitat availability and quality, and consequentially the population and productivity of marsh birds inhabiting them. To evaluate these relationships, we integrated long‐term, detailed geospatial metrics of multiple environmental stressors in tandem with field‐based hatching success data. Specifically, we investigated how multiple stressors and an extreme environmental perturbation (i.e., an 8‐year, record‐breaking lake level rise) impacted Black Tern population size and productivity at a historically large Black Tern colony (Wyman & Cuthbert, 2017) in St. Clair Flats between 2013 and 2020. During this period, Lake St. Clair's average lake levels increased by 1.03 m during the breeding season (May–July), comparable to historical extremes observed over longer time periods, including the maximum 10‐year decrease (−0.88 m from 1954 to 1964) and increase (1.25 m from 1964 to 1974) in the lake's water levels (Clites et al., 2014). Lake level increases of similar magnitude have also been recorded over 20‐year periods (1.17 m from 1819 to 1836) in Lake Michigan‐Huron (Quinn & Sellinger, 1990). We examined the relationship between biological and spatial habitat predictors and Black Tern hatching success and how these predictors changed with lake level rise. We then predicted changes in suitable breeding habitat extent and related these to breeding population size. Our work provides an example of how multiple stressors can reduce a region's ecological resilience to climate change‐related disturbance (i.e., extreme water level fluctuations) observed through coastal squeeze and subsequent loss of suitable breeding wetland habitat for Black Terns.

## MATERIALS AND METHODS

### Study region

St. Clair Flats is the largest inland freshwater delta in the world and covers roughly 101 km^2^ within Lake St. Clair. The region is historically home to one of the largest colonies of Black Terns in the Great Lakes (145–400 breeding pairs; Wyman & Cuthbert, 2017), and is attractive to Black Terns due to their preference for expansive wetlands alongside bodies of open water (Heath et al., 2020; Maxson et al., 2007). Because St. Clair Flats is capable of hosting a large Black Tern population and a diversity of other marsh bird species, it has been designated as a globally Important Bird Area (IBA) of high conservation value (Cheskey & Wilson, 2001). St. Clair Flats is dominated by native bulrush (*Schoenoplectus* sp.), cattail, and an invasive clonal dominant, common reed. Black Terns build nests almost exclusively from the broken stems of emergent vegetation in this region. Most nests sit on top of floating aggregations (mats) of these stems, though terns can also use logs, wood planks, or other floating debris.

### Monitoring population and hatching success

The Black Tern breeding colony at St. Clair Flats was monitored by a minimum of two people for at least 1–2 times per week between spring migration/arrival (~May 15) and fall migration (~July 30) from 2013 to 2020. Because Black Terns are semicolonial (Cuthbert, 1954; Heath et al., 2020), they often form smaller “sub‐colony” groupings dispersed throughout a wetland and will remain within the same relative location each year unless the sub‐colony is abandoned or the local habitat significantly changes. While a formal definition of a Black Tern sub‐colony in the literature is not readily available, we defined a sub‐colony here as a spatially distinct cluster of nests separated by physical gaps in nesting, major habitat changes, or other barriers (e.g., channels, open water, dense vegetation), where nesting pairs will exhibit distinct behavioral responses from other sub‐colonies (e.g., territorial mobbing is localized to the area). During the first week of each year, researchers identified any active or new sub‐colonies. For every sub‐colony visit, one researcher would approach the nesting area from the water (either on foot or kayak) until individuals from the sub‐colony flushed, and subsequently counted the number of birds (i.e., flush count) (Bracey et al., 2024; Zimmerman et al., 2002). Nests were monitored at 21 total unique sub‐colonies (average 9 per year), though the number of sub‐colonies with targeted nest monitoring varied by year depending on Black Tern breeding presence or absence. The estimated annual Black Tern population was based on the maximum flush count taken from all active sub‐colonies at the peak of the breeding season (mid‐June–July). For details, see Appendix S1.

Flush counts of sub‐colonies were additionally used to locate nests by identifying where adults took off or landed (Zimmerman et al., 2002). Once nests were found, they were georeferenced using a handheld GPS (2013–2016) or ArcGIS Collector App (2017–2020). Researchers visited each nest as frequently as possible during incubation (ideally at least once per week) to determine nest outcome, with a maximum of four visits (approximately every five days). Categories of nest outcomes were combined to determine whether nests either “failed” (*n* = 165), “hatched” (*n* = 286), or had an unknown fate (*n* = 370). In 2018 and 2020, data from field cameras (*n* = 18) helped inform nest failure, cause of failure, and hatch dates. Causes of nest failure were also recorded if they could be determined from field observations or nest camera footage. Nests were classified as failed if an adult was observed incubating on the previous visit, the nest was checked before the estimated hatch date, and breeding adults clearly no longer attended the nest. While this was often difficult to determine and documented as “unknown”, if the nest failed, researchers recorded the nest as (1) “disappeared entirely” and could not be relocated on the last visit or confirmed failed due to a weather event, (2) “washed away by a weather‐related event” if this was observed from nest camera footage or inferred if the nest was observed as washed away and experienced a 24‐h maximum lake level rise greater than 5 cm between visits (Clites et al., 2014), (3) “intact with missing eggs” if the nest itself was present but both eggs and breeding adults were not, (4) “predated” if eggshell fragments or punctured eggs were present, as camera footage and field observations confirmed that adults typically remove eggshells after hatching (Appendix S3; Cuthbert, 1954), or (5) “abandoned” if the eggs remained by breeding adults that were confirmed as no longer attending the nest. See Appendix S3 for further description of nest outcomes. The 95% confidence interval for each year's hatching success rate was calculated with a beta distribution using the qbeta() function from the “ExtDist” package in R (Wu et al., 2025).

### Number of nests within a 30‐m radius and clutch initiation date

We extracted two biological variables from nest monitoring data: number of nests within a 30‐m radius and clutch initiation date (date the first egg was laid). For the number of nests within a 30‐m radius of each individual nest, we used the spatial criteria for nesting “clusters” defined in Maxson et al. (2007). Rather than cluster size shared by all nests in a group, however, this metric represented a local neighborhood nest density and was quantified post‐field season using the “near” tool in ArcGIS Pro among nest GPS points from the same year. The clutch initiation date was determined by the following methods in order of highest to lowest accuracy: (1) observing the nest within the first 2 days of incubation, (2) adding the average incubation time (21–22 days; Heath et al., 2020) to when a chick was observed hatching, (3) adding incubation time to the estimated chick age based on chick mass, (4) applying an egg “float test” (Hays & Lecroy, 1971), or (5) estimating from fledgling age in rare circumstances when observations took place before and after fledging and the individual could be traced back to its nest of origin. If more than one of these observations were recorded, the method with the highest accuracy was applied. Additional information on methodological accuracy, selection, and age calculations are found in the Appendix S2.

### Relative initial nest water depth

Relative initial nest water depth was collected and calculated using a combination of 3‐m resolution bathymetry from the National Oceanic and Atmospheric Administration (NOAA) Office for Coastal Management Lake Level Viewer Dataset (Roth et al., 2016) and surface water levels from the NOAA Huron Erie Connecting Waterways Forecasting System (HECWFS) model (Clites et al., 2014). The bathymetric elevation (m) was determined for each nest in ArcGIS Pro using the “sample” function from the Lake Level Viewer Dataset (Roth et al., 2016). Water depths were then estimated by subtracting the bathymetric elevation from the HECWFS surface elevation, which was standardized to the lake level recorded at the Black Tern breeding season onset (May 15th). Estimated water depths were standardized to centimeters, with zero set as the shallowest relative depth. See Appendix S4 for more details.

### Fine‐scale geospatial habitat variables

We selected fine‐scale geospatial habitat variables based on previous literature on Black Tern habitat selection (see Appendix S5). These variables included: (1) distance between each nest and island developments (house distance) (m), (2) distance between each nest and the main lake (lake distance) (m), (3) distance to any open water (open water distance) (m), and (4) the percentage of four habitat classes within a 3‐, 7‐, and 12‐m radius of each nest. Habitat classes included open water and three vegetation types: sparse, medium, and dense vegetation. To calculate these metrics, we used 4‐band, <1‐m resolution images from multiple image sources. Open‐source aerial photographs from the National Agriculture Imagery Program (NAIP) were prioritized when available; however, years without NAIP images were supplemented with commercial satellite imagery, including Kompsat‐2, Triplesat‐3, and WorldView‐2 and 3. All images were taken within a nine‐day window between late June and July to minimize differences in seasonal vegetation phenology. Images were then resampled to 1‐m resolution using bilinear interpolation and geometrically, radiometrically, and atmospherically corrected based on their sensor requirements (Appendix S6). In preparation for image classification, normalized difference water index (NDWI) and normalized difference vegetation index (NDVI) rasters were derived from all 1‐m images. NDWI uses green and near infrared wavelengths to delineate water bodies (McFeeters, 1996), and NDVI applies red and near infrared wavelengths to measure photosynthetic concentrations, or “greenness” (Tucker & Sellers, 1986; Weier & Sellers, 2000). Details on NDWI and NDVI calculations are provided in Appendix S6.

Island developments, defined as houses, roads, docks, or any other permanent or semipermanent anthropogenic structures, were heads‐up digitized using 1‐m resolution NAIP imagery collected in 2014, 2016, and 2018. Open water was classified annually by applying Natural Breaks (Jenks) Unsupervised Classification on NDWI raster images. The main lake was defined as open water not fully enclosed by emergent vegetation and was generated annually by applying the ascending “boundary clean” tool in ArcGIS Pro. Additional details on classifying the main lake can be found in the Appendix S7. The distance (m) between each individual nest and island developments (house distance), any open water (open water distance), and the main lake (lake distance) during the associated year were extracted using the “near” tool in ArcGIS Pro.

Vegetation was classified into three categories: (1) sparse standing vegetation and/or sparse, scattered mat (sparse), (2) mat and medium density scattered or cut standing vegetation (medium), and (3) densely packed, standing vegetation (dense). Annual 1‐m NDVI raster images were masked to exclude open water and island development classes and then classified using the “iso cluster unsupervised classification” tool in ArcGIS Pro. To capture the percentage of open water and the three vegetation classes around each nest, each class was sampled within a 3‐m (similar to 2‐m in Maxson et al., 2007), 12‐m (Hickey & Malecki, 1997), and 7‐m (median of 2 and 12‐m) radius buffer using the “tabulate intersection tool”. Because high‐resolution imagery was unavailable in 2015, habitat class percentages for that year were estimated by averaging measured values from classified 2014 and 2016 maps (Appendix S6).

### Mean NDVI

Due to limited temporal availability of NDVI at <1‐m spatial resolution, we also quantified mean NDVI on a coarser spatial scale (<5‐m) as a proxy for emergent vegetation density. 4‐band images were obtained on the same date for each year from Rapideye‐5 and PlanetScope depending on availability. Images were resampled to 5‐m resolution using bilinear interpolation and geometrically, radiometrically, and atmospherically corrected following sensor requirements (Appendix S6). Mean NDVI values were sampled from a 7‐m and 12‐m buffer around each nest using the “zonal statistics 2” tool in ArcGIS Pro.

### Hatching success and habitat analysis

We used R version 4.0.3 (R Core Team, 2025) to analyze the relationships between hatching success and habitat variables. A binomial (logit‐link function) generalized linear mixed‐effects model (GLMM) using the glmer() function from the “lme4” package in R (Bates et al., 2015) was used to determine the influence of habitat and biological predictors on hatch success, and sub‐colony was included as a random intercept. To improve model convergence, we fit models using the glmerControl() function's “bobyqa” optimizer. The following 13 habitat or biological variables were examined in the analysis as fixed effects: (1) year, (2) average annual lake level during the breeding season (May–July), (3) number of nests within a 30‐m radius, (4) estimated start of incubation as the number of days before or after May 15th of each year (clutch initiation), (5) relative initial nest water depth (cm), (6) distance between each individual nest and island developments (house distance) (m), (7) distance between each nest and the main lake (lake distance) (m), (8) distance to any open water (open water distance) (m), (9) percent open water within a 3, 7, or 12‐m radius, (10) percent sparse vegetation within a 3, 7, or 12‐m radius, (11) percent medium vegetation within a 3, 7, or 12‐m radius, (12) percent dense vegetation within a 3, 7, or 12‐m radius, and (13) mean NDVI within a 7 or 12‐m radius. Other potential factors such as individual bird ID could not be incorporated due to lack of sufficient banding data. However, the prevalence of the same individuals across breeding seasons is likely relatively low due to the species' low site fidelity (Heath et al., 2020; Shephard et al., 2023). Continuous habitat variables were first checked for multicollinearity using a Pearson correlation test using the cor() function from the “DescTools” package in R (Signorell, 2025). Because vegetation cover variables were compositional and summed to 100%, we evaluated subsequent models using either percentage of dense vegetation alone, or the percentage of open water, sparse, and medium vegetation. To compare model coefficients, all continuous independent variables were normalized using the scale() function of the “base” package in R (R Core Team, 2025) which computes a z‐score for each variable using its mean and standard deviation. We then determined the appropriate spatial scale of either open water, sparse, and medium vegetation, or dense vegetation (3, 7, and 12‐m), and mean NDVI (7 and 12‐m), by initially fitting a candidate model for each radius with a consistent set of core predictors (number of nests within a 30‐m radius, clutch initiation, relative initial nest water depth, house distance, lake distance, and open water distance). We compared model fit using Akaike's information criterion (AIC) (Akaike, 2011) and predictive performance using the receiver operating curve (ROC) and area under the curve (AUC) using the roc() function from the “pROC” package in R (Robin et al., 2011). We selected the radius for the percentage of open water and vegetation classes and mean NDVI with the lowest AIC.

We then constructed a global model containing the following predictors: number of nests within a 30‐m radius, clutch initiation, relative initial nest water depth, house distance, lake distance, open water distance, either the percentage of dense vegetation or the percentage of open water, sparse, and medium vegetation within the selected radius, and NDVI within the selected radius. We also assessed two‐way interaction terms between initial nest water depth and lake distance. Candidate models were compared and refined using an all‐subsets regression using the dredge() function from the “MuMIn” package (Bartoń, 2022). Candidate models within a ΔAIC ≤2 of the top model were considered competitive. These models were further evaluated based on predictive performance (i.e., ROC and AUC). The final model retained ecologically relevant predictors and the lowest AIC. As a final diagnostic evaluation, we assessed whether hatch rate over time could be driven by changes in known nest fate by fitting a binomial general linear model (GLM) of the proportion of unknown nest survival as a function of year and weighted by annual sample size using the glm() function from the “stats” package (R Core Team, 2025).

### Annual changes in lake level and habitat analysis

To examine responses over time and their relationship to changing lake levels and habitat conditions, we plotted all habitat variables and clutch initiation dates included in the final GLMM against annual average May–July lake levels (Clites et al., 2014). To visualize changes in the geographic extent of the suitable nesting habitat, we applied the final GLMM to raster layers of spatial covariates corresponding to each predictor. To maintain consistency between the GLMM and spatial predictions, we standardized spatial covariates by applying the same mean and standard deviations. Because clutch initiation was not spatially explicit, this was included as a constant standardized mean value. We also incorporated sub‐colony as a random effect by extracting their conditional modes from the final GLMM using the ranef() function from the “lme4” package in R (Bates et al., 2015). For each year, we calculated the linear predictor of each raster cell by taking the sum of the fixed‐effect intercept, the product of the fixed‐effects coefficients and standardized raster covariates, and the rasterized sub‐colony random intercept. We then calculated the hatching success probabilities for each cell by applying the inverse logit transformation to the linear predictor (Appendix S8).

We masked final maps to exclude regions that were determined a priori to be unsuitable for nesting. This included developed or dryland islands and peninsulas, open water, and areas where vegetation within a 7‐m radius exceeded an NDVI value of 0.72. Specifically, 0.72 was the maximum NDVI value surrounding a known nest, and it is assumed that the likelihood of any nest existing within areas with any higher value is extremely low.

To quantify sub‐colony breeding pair population size and their response to changes in sub‐colony habitat in the geospatial model, we applied a second GLMM using the lmer() function from the “lme4” package in R (Bates et al., 2015). Sub‐colony was included as a random intercept to account for repeated observations at the same location across multiple years (*n* = 13). However, we did not investigate interannual variation due to uneven and limited repeated visits across sub‐colonies. We chose predictor variables a priori, and included the area of open water, area of uninhabitable vegetation (NDVI >0.72), area of habitable vegetation, and area with >50% hatch success. We selected the area with >50% hatch success as this likely represents greater habitat suitability and because Black Terns are more likely to return to stable, productive locations (e.g., Atamas & Tomchenko, 2020). The area of classified predictor variables was extracted from the geospatial model outputs in ArcGIS Pro for sub‐colonies that contained clearly defined population counts and geospatial coverage in the same year for at least 2 years (*n* = 13). The response variable, that is, the number of maximum breeding pairs per sub‐colony, as well as the predictor variables were scaled using the scale() function from the “base” package in R (R Core Team, 2025) prior to running the GLMM, to account for considerable differences in measurement units. The area with >50% hatch success and habitable vegetation were correlated and were evaluated with the remaining predictors separately. The selected model was chosen based on having the lowest AIC and highest *R* ^2^.

## RESULTS

### Hatching success & habitat impacts

During 8 years of field monitoring hatch rates and population counts in the St. Clair Flats region (2013 to 2020), Lake St. Clair's average May–July lake level (during the Black Tern breeding season) rose by 1.03 m (0.14 m/year) as the result of regional changes in climate (Clites et al., 2014) (Figure 2). Hatch rate (percentage of hatched nests out of nests of known status) dropped by 55% between 2013 (90%) and 2020 (35%) with an annual average decline of 7.93% (Figure 2). After applying a binomial GLM of the proportion of unknown nest survival weighted by annual sample size, we found that hatch rate over time was not driven by changes in known nest fate. The proportion of unknown survival (percentage of nests with an unknown status out of all known and unknown status) varied 28–66% across years and showed no significant temporal trends (β = −0.023 ± 0.037 SE, *z* = −0.62, *p* = 0.53), and an estimated annual decline of 0.57%. Corresponding with the decline in hatch rate, the monitored population of Black Terns at Lake St. Clair Flats dropped by 77% since 2013, on average by 22.16 pairs/year (Figure 2). The causes of nest failure varied in rate and detectability where 35.48% were unknown, 32.25% of failed nests disappeared entirely, 15.32% were likely washed away during a weather‐related event, 7.25% had an intact nest with missing eggs, 6.45% were predated, and 3.22% were abandoned. In four cases, chicks were found dead from an unknown cause.

**FIGURE 2:**
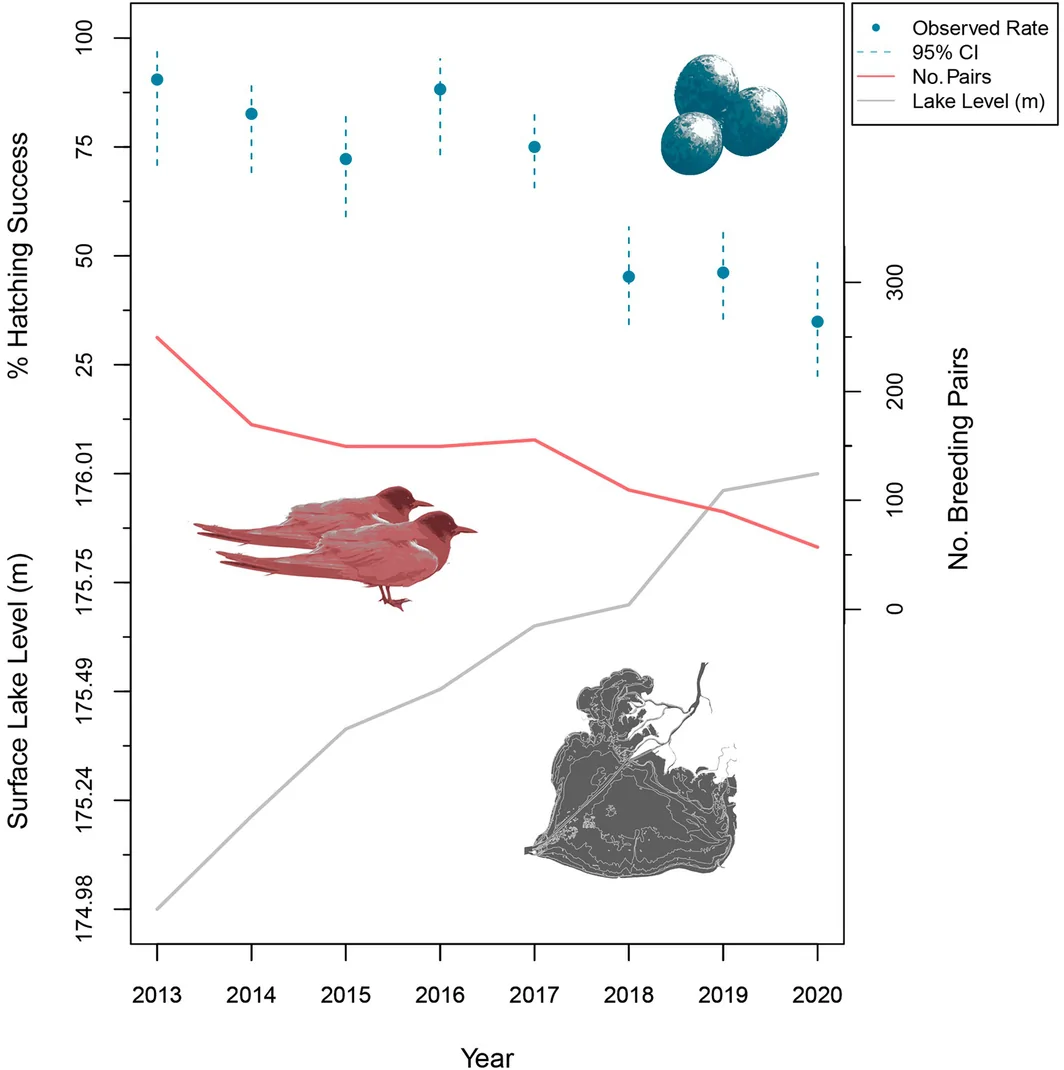
Yearly estimated number of Black Tern breeding pairs across St. Clair Flats (solid red line, right *y*‐axis), average surface lake level (May–July) (m), and yearly hatching success rates (% hatched of total nests and 95% confidence intervals (CI), dots, and whiskers, left *y*‐axis). Population size is a “best” estimate of individuals, with maximum and minimums (shown by shaded sections) recorded in 2013 and 2014. Credit for Black Tern eggs (top right) and Black Tern adult pair (center): Jennifer L. Fuller. Credit for bathymetric map (bottom right): Jennifer L. Fuller (based on data from Roth et al., 2016).

We applied a stepwise binomial (logit‐link function) GLMM to compare 244 hatched nests and 126 failed nests, with sub‐colony included as a random effect (*n* = 18). Year, average annual lake level during the breeding season, and relative initial nest water depth were correlated (*r*_s_ > 0.6). We retained relative initial nest water depth as this was more likely to have a direct impact on hatching success. No other r_s_ values were greater than 0.6, and all remaining predictors were retained. Model selection favored habitat covariates measured at a 12‐m radius for percent open water and vegetation, and a 7‐m radius for mean NDVI, minimizing AIC, and maximizing AUC values (Appendix S10). We also chose the percentage of dense vegetation instead of a combination of open water, sparse, and medium vegetation as this reduced AIC and simplified the overall model.

All of our candidate models (ΔAIC <3) contained the same set of base predictors: clutch initiation, relative initial nest water depth, lake distance, mean NDVI (7‐m radius), and % dense vegetation (hereafter, “base model”) (Table 1). The top two candidate models included the base model + open water distance and the base model alone, with a ΔAIC of 0 and 0.8, respectively. As either model had similar support, we chose the base model + open water distance as the final model. Although the effect of open water distance was marginal, it was retained due to its plausible ecological influence on hatching success not already captured by other predictors. No interaction terms were retained, and the final model exhibited high predictive performance (AUC = 0.84). Results of the final selected model are summarized in Table 2 and Figure 3. Hatching success was negatively and significantly (alpha <0.05) related to the date of clutch initiation, relative initial nest water depth and the percentage of dense vegetation within a 12‐m radius but positively related to lake distance and mean NDVI within a 7‐m radius. Hatching success was negatively related to open water distance, but this relationship was not statistically significant. Later clutch initiation dates and increasing amounts of dense vegetation consisting of common reed around a nest were negatively associated with hatching success. The average percentage of dense vegetation surrounding a nest's 12‐m radius was 12% lower in hatched versus failed nests (14% vs. 26%). Excluding outliers beyond 1.5 times the interquartile range, the maximum percent cover of dense vegetation for hatched nests was less than 45%, whereas the maximum for failed nests reached 93%. Relative initial nest water depths were on average 13.42 cm shallower in hatched nests than those that failed. Hatched nests were found to have a mean distance of 39.71 m from the open water of the main lake, while failed nests were moderately closer (21.71 m). The mean percentage of NDVI in a 7‐m radius in hatched nests was slightly higher (0.36) than failed nests (0.32). Though open water distance was not a significant predictor of hatching success, more successful nests were on average 2.14 m closer to open water patches than failed nests.

**TABLE 1:** Summary of candidate generalized linear mixed‐effects model (GLMM) results predicting hatching success.

Top candidate models explaining hatching success			
Predictors	AIC	ΔAIC	AUC
**base + open water distance**	**398.07**	**0**	**0.843**
Base	398.87	0.8	0.839
base + house distance + open water distance	399.04	0.97	0.844
base + house distance	399.71	1.65	0.84
base + No. nests within a 30‐m radius + open water distance	399.86	1.79	0.844
base + No. nests within a 30‐m radius + house distance + open water distance	400.65	2.58	0.846
base + No. nests within a 30‐m radius	400.74	2.68	0.841

*Note*: The ‘base’ model contains the following predictors included in all candidate models: Clutch initiation, relative initial nest water depth, lake distance, mean NDVI (7‐m radius), and % dense vegetation, with sub‐colony included as a random effect. The table reports the best‐supported model output from each model input combination, ranked by Akaike's information criterion (AIC). Δ AIC values are calculated relative to the top‐ranked model across all model combinations, and model performance is summarized as the receiver operating characteristic curve (AUC). The final model selected based on the lowest AIC is indicated in bold.

**TABLE 2:** Summary table of the selected binomial generalized linear mixed‐effects model (GLMM) results to predict hatching success in response to biological and environmental predictors.

Effects of biological and environmental predictors on hatching success				
Model component	Parameter	Coefficien*t*	CI	*p*
Fixed effects	Intercept	1.157	0.49–1.83	0.001
	Clutch initiation	−0.444	−0.71 to −0.18	**0.001**
	Relative initial nest water depth	−0.374	−0.69 to −0.06	**0.019**
	Lake distance	0.539	0.17–0.90	**0.004**
	Open water distance	−0.332	−0.71–0.04	0.083
	% Dense vegetation (12‐m radius)	−0.597	−0.91 to −0.29	**<0.001**
	Mean NDVI (7‐m radius)	0.599	0.17–1.03	**0.007**
Random effect	τ_00 sub‐colony_	1.29	…	…
Model statistics	ICC	0.281	…	…
	*N*_sub‐colony_	18	…	…
	Observations	370	…	…
	Marginal *R* ^2^	0.249	…	…
	Conditional *R* ^2^	0.46	…	…

*Note*: Includes predictor coefficients, 95% confidence intervals (CI), and *p*‐values (*p*) for fixed effects. Coefficients for additional parameters, including between‐subjects variance (τ_00 sub‐colony_), interclass correlation coefficient (ICC), number of sub‐colonies sampled (*N*), number of observations, and variance explained by fixed effects (Marginal) and fixed and random effects (Conditional) *R* ^2^ are also reported. Significant *p*‐values (alpha <0.05) are indicated in bold.

**FIGURE 3:**
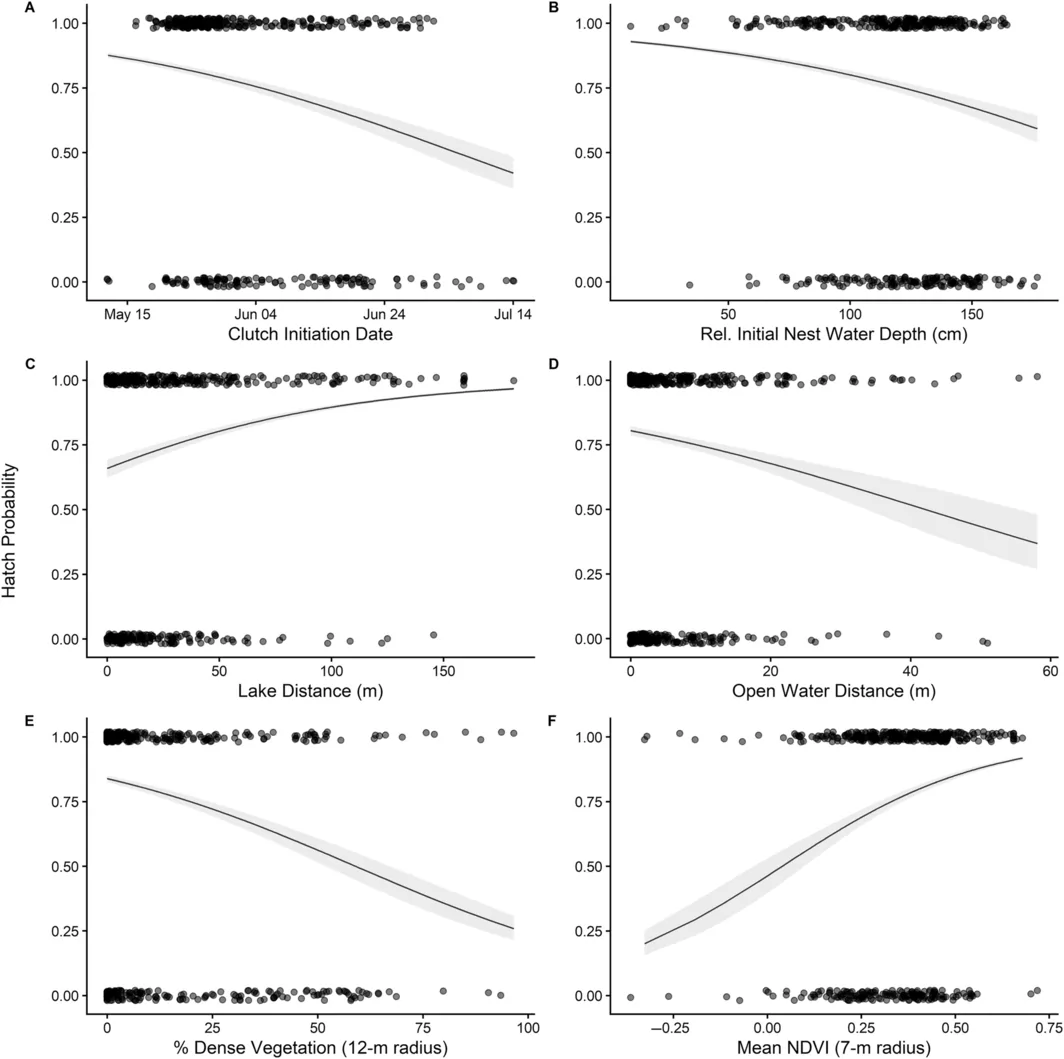
Relationships between hatching success probability and biological and environmental predictors (A–F) included in the final binomial generalized linear mixed‐effects model (GLMM) including sub‐colony as a random intercept. Points represent nest outcomes (0 = failed, 1 = hatched), the solid line represents predicted probabilities, and the shaded area represents 95% confidence intervals. Predictors were scaled for analysis but presented on their original scales for interpretability.

### Yearly responses to lake level & habitat change

When we plotted habitat variables and clutch initiation dates against yearly average May–July lake levels (Figure 4), average relative initial nest water depth increased steadily as annual lake levels rose, but the average distance between nests and open water of the main lake fluctuated between years without showing particular trends. The extent of dense vegetation surrounding nests remained consistently low until 2017, when the lake level reached a height of 175.65 m above sea level (asl.) and birds were apparently forced to shift their nests into denser, less favorable vegetation. Clutch initiation dates did not show any significant trends with increasing lake level except for the last two study years, when start dates appeared to extend substantially later into the summer season. Average NDVI did not vary in a predictable manner over the course of the study. With the exception of 2013, nests were closer to open water after 2017 than previous years, which is consistent with the effects of lake level attenuating emergent vegetation.

**FIGURE 4:**
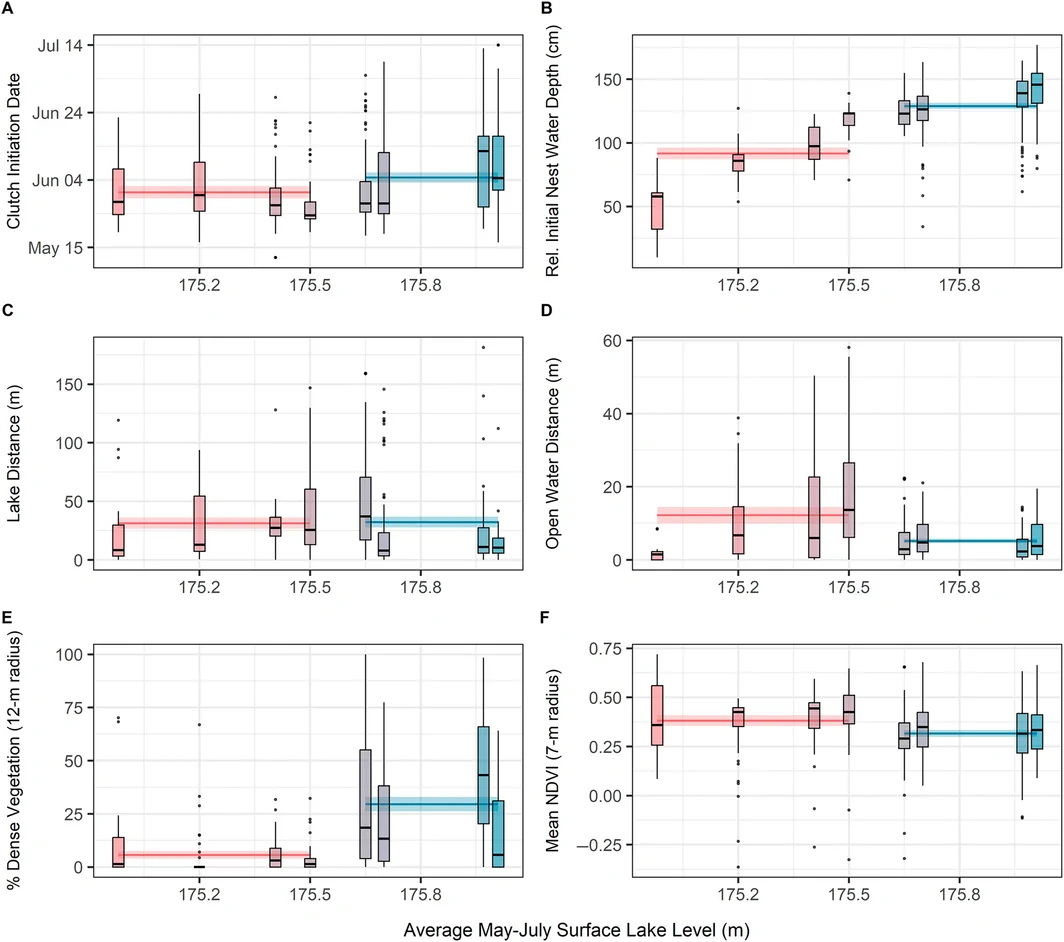
Boxplots of biological and environmental predictors (A–F) included in the final binomial generalized linear mixed‐effects model (GLMM), against the respective average May–July surface lake level. Lake levels increased near monotonically over the course of the study. The average median for values from 2013 to 2016 (red) and 2017–2020 (blue) are plotted as lines for reference with respective 90% confidence intervals.

To investigate the relationship between nesting habitat availability and population size, we developed a GLMM predicting the annual maximum number of breeding pairs at 13 sub‐colonies between 2013 and 2020 (Table 3). Wetland categories were derived from the geospatial output (e.g., Figure 5). Figure 6 illustrates the results of the GLMM which investigates sub‐colony population size as a response to changes in two major wetland categories derived from the spatial model. These two categories include the area of habitat with >50% hatch success and open water. The number of breeding pairs was significantly related to the sub‐colony area with >50% hatch success (*p* < 0.001) (Table 3, Figure 6A). Area of open water was not a significant predictor of the number of sub‐colony breeding pairs, though it improved the model's marginal and conditional R^2^ (0.403, 0.456, consecutively) compared to the model with only >50% hatch success.

**TABLE 3:** Summary table of the generalized linear mixed‐effects model (GLMM) predicting number of breeding pairs by changes in scaled area of two land cover classifications based on nesting habitat quality or habitability: >50% hatch success and open water regions.

Effects of land cover area on number of breeding pairs				
Model component	Parameter	Coefficient	CI	*p*
Fixed effects	Intercept	−0.010	−0.26–0.24	0.937
	Area with >50% hatch success (scaled)	0.612	0.38–0.84	**<0.001**
	Area of open water (scaled)	−0.254	−0.49 to −0.01	0.053
Random effect	τ_00 sub‐colony_	0.1	…	…
Model statistics	ICC	0.09	…	…
	*N*_sub‐colony_	13	…	…
	Observations	49	…	…
	Marginal *R* ^2^	0.403	…	…
	Conditional *R* ^2^	0.456	…	…

*Note*: Includes predictor coefficients, 95% confidence intervals (CI), and *p*‐values (*p*) for fixed effects. Coefficients for between‐subjects‐variance (τ_00 sub‐colony_), interclass correlation coefficient (ICC), number of sub‐colonies sampled (*N*), number of observations and variance explained by fixed effects (Marginal), and fixed and random effects (Conditional) *R* ^2^ are also reported. Significant *p*‐values (alpha <0.05) are indicated in bold.

**FIGURE 5:**
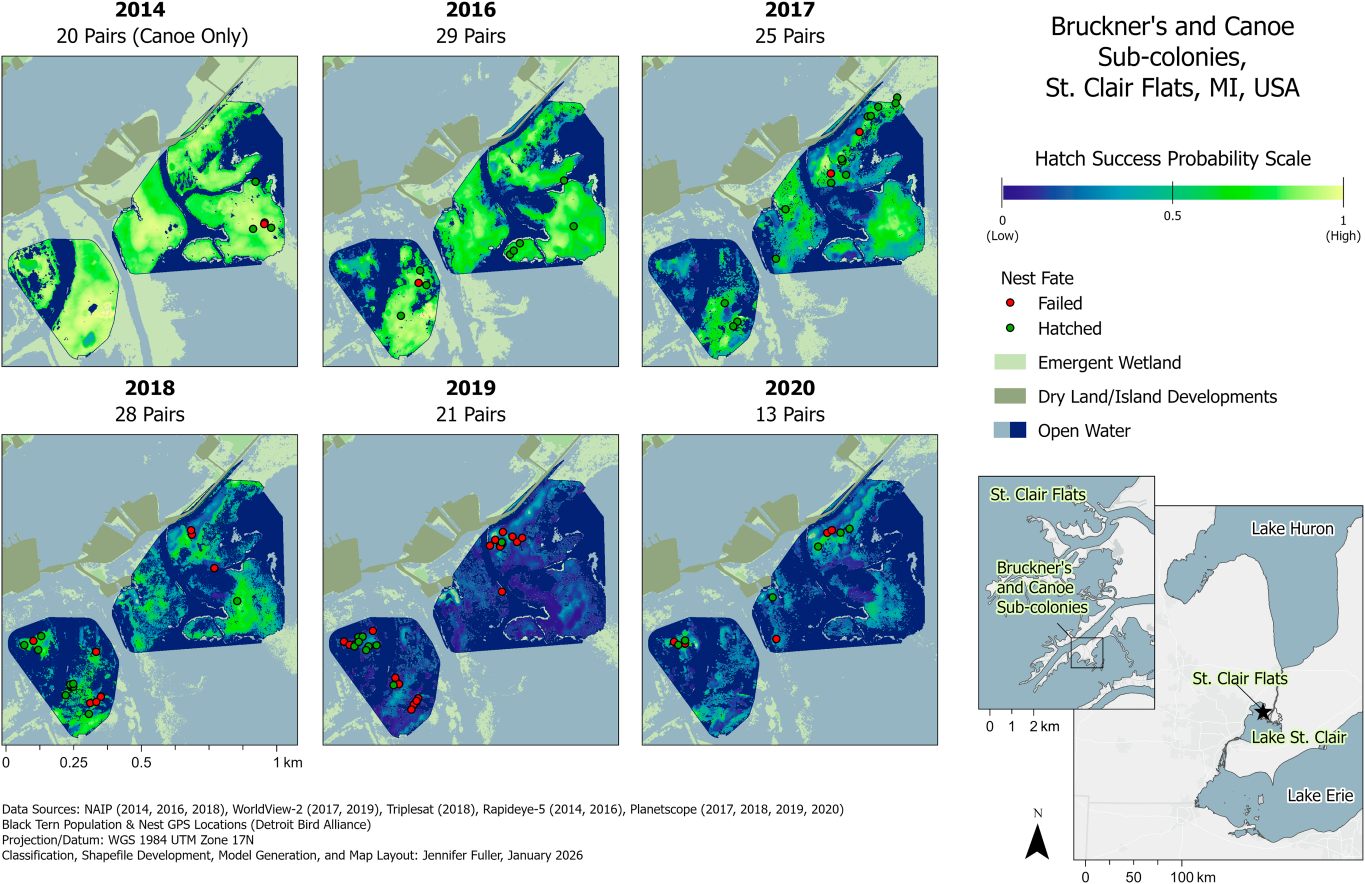
Map of geospatial hatch success output of two example sub‐colonies in the St. Clair Flats, MI, USA, showing decline in area of hatching success in 2014 and 2016–2020. Modeled hatching success was derived from predictors using the final binomial generalized linear mixed‐effects model (GLMM). Polygons delineate two sub‐colonies: Bruckner's (left) and Canoe (right). Polygons only include areas with full imagery data across years; therefore, 2013 and 2015 were excluded.

**FIGURE 6:**
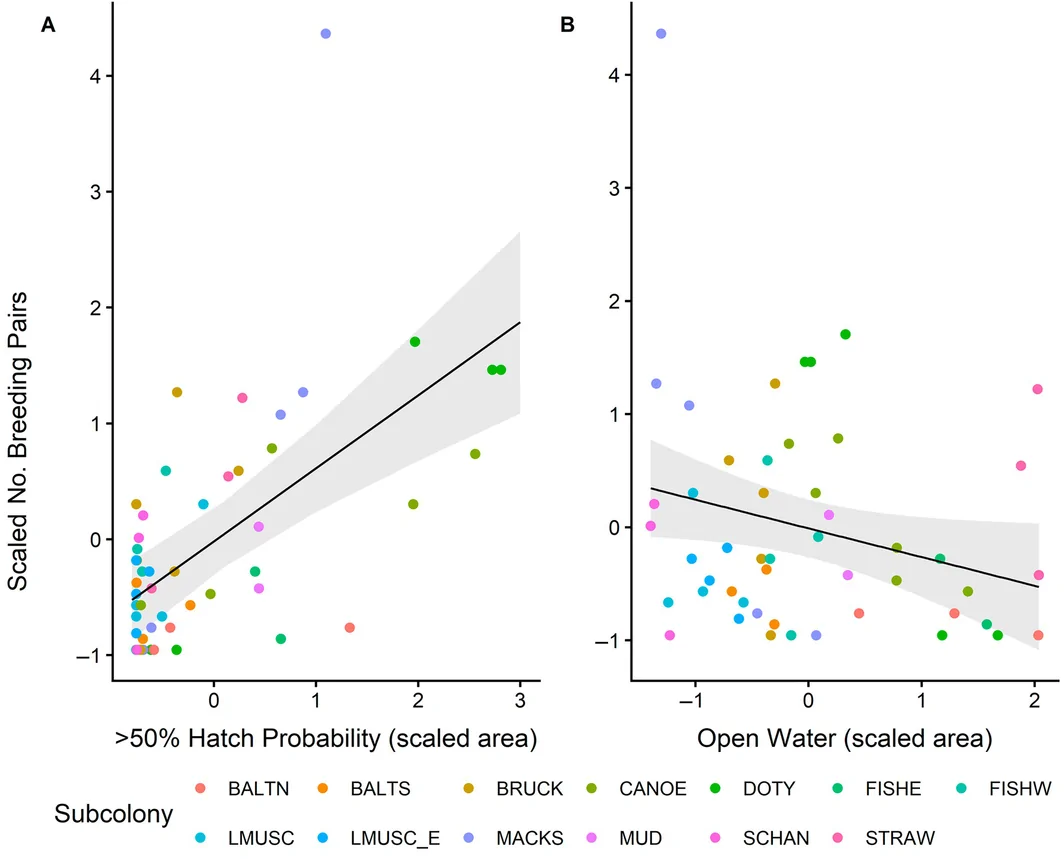
Generalized linear mixed‐effects model (GLMM) plots predicting the response of number of breeding pairs by changes in scaled area of two land cover classifications based on nesting habitat quality or habitability, with sub‐colony included as a random effect: (A) >50% hatch success and (B) open water. Colored points denote the scaled number of breeding pairs versus the scaled area of each land cover class. Model estimates and 95% confidence intervals are represented by the solid line and filled area, consecutively.

## DISCUSSION

In our study, we addressed two goals: (1) to understand how multiple stressors may limit ecosystem resilience in the context of climate change through breeding bird response and (2) to clarify the implications of these findings on coastal wetland and avian management in the Great Lakes region. The drivers of nest failure and colony abandonment in a freshwater coastal wetland included deeper water at the nest site, closer proximity to the turbulent open water area of the main lake, and denser surrounding vegetation. Overall, our results show how multiple stressors erode coastal wetland resilience to extreme lake level rise, as indicated by a sharp decline in Black Tern breeding population presence and hatching success. While we were unable to compare historical population responses to similar lake level changes prior to invasive species establishment and human development, this substantial decline in productivity and suitable breeding habitat suggests a rapid structural and functional change in the ecosystem that potentially exceeds natural variability. This change reflects a shift towards an alternative state unable to support historical breeding population sizes and productivity.

### Clutch initiation

To understand what may influence the decline of Black Tern populations, it is important to consider what factors may affect productivity. Similar to previous studies of Black Terns (Golawski & Mroz, 2019) and other seasonally breeding birds (Perrins, 1970; Verhulst & Nilsson, 2008), we found that earlier clutch initiation was significantly correlated with improved hatching success. Although we did not observe a clear relationship between clutch initiation timing and annual lake level trends, nests in 2019 were laid considerably later than in other years, and clutch initiation dates in 2019 and 2020 exhibited higher variability when lake levels were at their highest (Figure 4). Reduced high‐quality nesting habitat availability due to high water level extremes could increase nesting site competition, time required to locate suitable nesting habitat (Shealer et al., 2006), and late‐season renesting attempts due to deteriorating habitat and increased nest failure rates.

### Dense vegetation and NDVI

The amount of surrounding habitat in the densest vegetation category was the strongest predictor of nest failure, which was predominantly composed of invasive common reed (Bourgeau‐Chavez et al., 2019; Appendix S9), while in contrast, mean NDVI had a positive effect on hatching success. Tall and dense growth patterns of common reed and cattail (Swearingen et al., 2012) likely reduce breeding adults' ability to raise young successfully by impairing takeoff, landing, access to nests (Maxson et al., 2007), and predator detection (Firstencel, 1987; Moy, 1995). Dense vegetation may also increase nest proximity to predator denning and diurnal resting areas (e.g., raccoons and striped skunks, Peterson et al., 2022) and/or provide platforms that facilitate terrestrial predator access. Repeated field observations also suggest that dense growth patterns of common reed and cattail may limit the accumulation of high‐quality floating mats for nesting, whereas more dispersed vegetation such as bulrush may be more conducive to mat formation. However, these observations were not formally tested and would benefit from evaluation using imagery with higher spatial resolution (e.g., from drones). In contrast to dense vegetation, there was a positive relationship between hatching success and mean NDVI within a 7‐m radius, though the difference between the average NDVI and failed and successful nests was slight. NDVI was not correlated with dense vegetation and may be representative of microhabitat with a relatively higher mix of vegetation types and/or mat availability, providing greater nest concealment from predators, nest stability, and protection from wind and waves (Albert et al., 2005; Mosher, 1986). Finally, closer proximity to open water patches, while not significant in our model, could improve hatching success by creating barriers to land‐originating predators or indicating greater local habitat interspersion.

Climate‐driven water level fluctuations such as severe drought can facilitate the encroachment of dense, invasive vegetation, which has resulted in widespread Black Tern colony abandonment in the Great Lakes (Wyman & Cuthbert, 2017) and the Prairie Pothole region (Naugle et al., 2000). Conversely, extreme high water levels coupled with established dense vegetation and hardened shorelines can result in flooding of the preferred patches of short, dispersed vegetation (e.g., bulrush), and prevent the accumulation of high‐quality nesting mats (Bergman et al., 1970; Bourgeau‐Chavez et al., 2015; Craig & Beal, 1992; Cuthbert, 1954; Gnass Giese et al., 2018; Heath, 2004; Heath et al., 2020; Mosher, 1986; Prince et al., 1992; Weller & Spatcher, 1965; Wilcox, 2012), a process known as “coastal squeeze” (Anderson et al., 2023; Pontee, 2013; Tozer et al., 2024).

### Water depth and distance to the main lake

We found that increased relative initial nest water depth and proximity to the main lake negatively impacted hatching success. The fragility and low‐lying proximity of Black Tern nests to water increases their vulnerability to wind, waves, and water fluctuations (Bergman et al., 1970; Gilbert & Servello, 2005; Mosher, 1986), as further evidenced by our finding that 15% of nests failed following a 24‐h maximum lake level rise exceeding 5 cm. While increased proximity to larger expanses of open water (e.g., the “main lake”) may discourage land‐originating predators, similar to smaller open water patches, predator accessibility may still be high if a nest is also situated alongside dense vegetation. This potential benefit is also likely outweighed by greater exposure to higher energy wind, waves, and currents augmented by deeper water, increased fetch (i.e., distance wind travels over open water to generate waves), reduced vegetative buffering (Albert et al., 2005; Mortsch et al., 2006), and increased boat traffic. Vegetated wetlands can buffer nests from wind and wave action and reduce failure risk. However, extreme lake level rise, especially when coupled with coastal squeeze, can flood and attenuate emergent vegetation, destabilize or wash away floating mats, and increase the proximity and exposure of nesting areas to higher energy lake conditions (Albert et al., 2005; Mosher, 1986). Although additional data identifying the proximate causes of nest failure are needed, nest camera evidence suggests that storm‐driven wave action coupled with lake exposure likely contributed to increased nest failure (Appendix S1). Other mechanisms associated with deeper water (e.g., disturbance via common carp; Lougheed et al., 1998, Hennen & Brown, 2014; Appendix S1) may also contribute to habitat degradation and nest failure risk, though these factors require further investigation.

### Local landscape changes and coastal squeeze

Our geospatial models suggest that rising lake levels reduced the availability of favorable nesting habitat, clarifying the observed declines in hatching success and colony abandonment. Following a period below‐average lake levels (1999–2013), Lake St. Clair experienced a steady increase of 1.03 m between 2013 and 2020 (Clites et al., 2014). Our geospatial outputs confirmed these increases corresponded with a marked transition from favorable habitat of patchy emergent vegetation (i.e., >50% hatch success probability) to more hazardous areas characterized by deeper waters, dense vegetation, and increased proximity to the main lake. As expected, the extent of habitat with >50% hatch success probability was a significant predictor of sub‐colony population size. As this favorable habitat declined, other indirect pressures may have further reduced hatching success, such as increased habitat overlap and nest‐trampling risk by other species (e.g., American Coot (*Fulica americana*), Common Gallinule (*Gallinula galeata*), Mallard (*Anas platyrhynchos*), and muskrat (*Ondatra zibethicus*)), as well as increased predation risk from land‐originating nest predators such as eastern fox snake, American mink (*Neovision vision*), and raccoon (*Procyon lotor*). Habitat loss may have increased predator accessibility to nests by reducing search area and increasing surrounding dense vegetation (Heath, 2004).

Our study exemplifies the phenomenon of “coastal squeeze”, in which wetland habitat declines as a result of lake level rise and with the formation of barriers to landward migration (Anderson et al., 2023; Pontee, 2013; Tozer et al., 2024). Resilient marsh communities respond to hydrologic changes by migrating lakeward during receding lake levels and advancing shoreward during rising lake levels, provided the condition in either direction is habitable (e.g., hydric soil condition, water depth, hydrologic energy) (Bansal & Lishawa, 2019; Wilcox, 2004). This relationship between vegetation and water level maintains habitat heterogeneity and reduces monocultural growth, which in turn supports marsh bird breeding and foraging (Bansal & Lishawa, 2019; Craig & Beal, 1992; Kantrud & Stewart, 1984; Wilcox, 2004). However, increased “hardening” of the shoreline through human development prevents shoreward advancement of marshland communities during high lake levels. This loss of wetland adaptive capacity due to shoreline hardening is a pervasive issue in the Great Lakes (Wensink & Tiegs, 2016) and worldwide (Dugan et al., 2011).

In St. Clair Flats, geospatial outputs suggest that favorable nesting habitat did not shift shoreward with rising lake levels, which could have been partly due to the presence of shoreline development and common reed (e.g., Figure 5). Continuously low lake levels from 1999 to 2013 facilitated the propagation and subsequent aggressive expansion of common reed along the coastal margin, where it advanced clonally into submerged soils and persisted through lake level rise up to depths of 1.8 m (McFeeters, 1996; Wilcox, 2012; Wyman & Cuthbert, 2017). The presence of common reed in turn restricted the shoreward movement of favorable, patchy emergent communities such as bulrush, resulting in inundation at the deeper end of their distribution. The expansion of invasive cattail (*T. × glauca* and *T. angustifolia*) in the Great Lakes between 2011 and 2013 (Carson et al., 2018) likely posed a similar threat to Black Terns and freshwater wetland biodiversity. Invasive cattail species also exhibit competitive dominance and tolerate hydrologic extremes (Bansal & Lishawa, 2019); however, common reed was significantly more widespread in St. Clair Flats and largely avoided by Black Terns. Simply put, coastal squeeze contributed to the loss of diverse, productive habitat for Black Terns and ultimately, colony persistence.

### Basin‐wide historical context

A review of historical and basin‐wide conditions illustrates the complex and context‐dependent role lake level fluctuations play in Black Tern habitat availability and population trends. The Breeding Bird Survey (BBS) showed that between 1967 and 1999, Black Tern populations throughout the Great Lakes (total population surveyed in Ontario, Canada, and all Great Lakes States; MI, OH, IN, MN, IL, PA, NY) declined at a rate of roughly 0.89% per year (Pardieck et al., 2020). Lake levels during this period were variable (maximum decadal changes of 0.47 m and −0.64 m, Lake Huron‐Michigan) and generally above‐average (Quinn & Sellinger, 1990). During the following drought period with below‐average lake levels (1999–2013) (Clites et al., 2014; Quinn & Sellinger, 1990), this rate increased to 2.41% loss per year (Pardieck et al., 2020). BBS surveys took place in both coastal and inland wetland locations, and in these locations, Black Terns faced a myriad of potential factors leading to their decline. However, large‐scale studies on a variety of coastal wetlands largely found that several marsh birds (e.g., Black Tern, American Bittern (*Botaurus lentiginosus*), Least Bittern, American Coot, Sora) populations declined due to drought‐enabled encroachment of invasive vegetation (Mortsch et al., 2006; Timmermans et al., 2008; Wyman & Cuthbert, 2017). Between 1996 and 2013, Black Tern populations fell 4.9% annually in the Southern Great Lakes Basin (Tozer, 2015), and populations across the Great Lakes coastal wetlands declined by 42% between 1999 and 2009 (Cuthbert & Wires, 2013). Populations and site occupancy in Wisconsin declined by around 70% between 1980 and 2011 as a result of breeding habitat loss and low adult survival. In Green Bay, Wisconsin, for example, population losses were attributed to common reed invasion overtaking 75% of viable breeding habitat between 1985 and 2002 (Matteson et al., 2012). Additionally, between 1976 and 2009, increased dense vegetation contributed to 90% abandonment of 78 Black Tern colonies in the Great Lakes. Lake levels posed an unclear but likely indirect influence by altering vegetation structure (Wyman & Cuthbert, 2017).

As lake levels rose after 2013, local conditions continued to influence adaptability of important marsh bird and Black Tern habitat. From 2013 to 2017, rising lake levels positively correlated with marsh bird populations (including Black Tern) in coastal Green Bay, Wisconsin. These rising lake levels may have revitalized preferred patchy emergent vegetation in this location (Gnass Giese et al., 2018). However, the study did not continue after 2017 when conditions for Black Terns worsened at Lake St. Clair. Unlike Green Bay, Lake St. Clair's Black Tern colony was at its known healthiest population and breeding success rates in 2013 and 2014 but declined with rising lake levels until 2020. Another study conducted between 2011 and 2021 attributed greater Black Tern populations to increasing lake levels in the Great Lakes; however, surveys were limited to Lake Huron and Lake Erie (Tozer et al., 2024). On average, these coastal colonies may be more resilient to higher lake levels than St. Clair Flats and improve the regional portfolio of habitat resilience and availability. However, Tozer et al. (2024) emphasizes that further increases in lake levels could result in increased instances of “coastal squeeze”, reducing the capacity of Great Lakes coastal wetlands to support regional marsh bird populations, including Black Terns. This highlights the context dependence of wetland resilience and the importance of comprehensive, basin‐wide understanding of wetland response to different climate scenarios.

### Conservation and management implications

Considering the variability of stressors and environmental characteristics of individual wetlands (Mortsch et al., 2006) and applying comprehensive assessments across diverse locations (e.g., Great Lakes Coastal Wetlands Monitoring Program; Uzarski et al., 2017) could enhance our understanding of the Great Lakes' wetland resilience to climate change. Effective restoration will require tailoring conservation efforts to habitat‐specific characteristics (Mortsch et al., 2006), forecasting the interaction of stressors under different climate scenarios (Reid et al., 2018; Staudt et al., 2013), and considering the regional portfolio of breeding locations (Hanski & Simberloff, 1997; Matthiopoulos et al., 2005). Many species, including the Black Tern, can adapt to spatiotemporal variability in wetland habitat via dispersal (Derosier et al., 2015; Johst et al., 2002; Sauer et al., 2014). However, land cover changes, invasive species, and hydrologic extremes may disrupt the natural turnover of suitable wetlands that support healthy metapopulations (Tulbure & Johnston, 2010). Between 1991 and 2009, targeted surveys observed a 90% decline in Great Lakes Black Tern nesting colonies (Wyman & Cuthbert, 2017), and a significant portion of breeding habitat is predicted to decline 97% by 2100 (Steen & Powell, 2012). Our findings underscore the extent to which wetland loss and multiple stressors may be limiting the species' resilience, and the need for a networked regional conservation approach that protects and restores large, diverse wetland areas with differing hydrological vulnerability. A broader portfolio of protected wetlands may create asynchronous habitat availability, ensuring suitable nesting sites persist even under fluctuating conditions (Aplet & McKinley, 2017; Hanski & Simberloff, 1997; Matthiopoulos et al., 2005).

Our study exemplifies a potentially synergistic interaction of multiple stressors (e.g., human development and invasive species) in conjunction with local environmental factors (e.g., bathymetry, biodiversity) that reduced ecosystem resilience to climate‐driven lake level changes (i.e., coastal squeeze). By integrating remote sensing to identify environmental stressors, long‐term field surveys to assess biological responses, and geospatial modeling to analyze spatial‐scale impacts, we provide a methodology for assessing wetland changes and guiding basin‐wide conservation efforts for vulnerable species. Prioritizing metapopulation‐scale planning and restoration to promote resilience may prevent conservation bottlenecks that arise when management efforts focus too narrowly on a few priority sites with limited resilience to climate extremes. Our findings highlight that even the largest historical reservoirs of biodiversity can collapse when climate extremes are compounded with habitat degradation, invasive species, and development. Conservation strategies for wetland‐dependent species should prioritize disseminating actionable conservation strategies, protecting a diverse regional portfolio of critical habitats, and employing resilience‐driven restoration to mitigate ongoing and future threats posed by climate change‐mediated local and regional stressors.

## AUTHOR CONTRIBUTIONS

Conceptualization: Jennifer L. Fuller, Erin Ford, Karen M. Alofs, Johannes Foufopoulos; Methodology: Jennifer L. Fuller, Karen M. Alofs, Johannes Foufopoulos, Andrew Gronewold; Investigation: Jennifer L. Fuller, Ava Landgraf, Erin Ford; Visualization: Jennifer L. Fuller, Andrew Gronewold; Funding acquisition: Jennifer L. Fuller, Karen M. Alofs; Project administration: Karen M. Alofs, Erin Ford; Supervision: Karen M. Alofs, Johannes Foufopoulos, Andrew Gronewold; Writing original draft: Jennifer L. Fuller; Writing review & editing: Jennifer L. Fuller, Karen M. Alofs, Johannes Foufopoulos, Andrew Gronewold, Erin Ford, Ava Landgraf.

## CONFLICT OF INTEREST STATEMENT

The authors declare no conflicts of interest.

## Supporting information


Appendix S1.

Appendix S2.

Appendix S3.

Appendix S4.

Appendix S5.

Appendix S6.

Appendix S7.

Appendix S8.

Appendix S9.

Appendix S10.

## Data Availability

Data and code (Fuller et al., 2026) are available in Zenodo at https://doi.org/10.5281/zenodo.19290242.

## References

[eap70314-bib-0001] Akaike, H. 2011. “Akaike's Information Criterion.” In International Encyclopedia of Statistical Science, edited by M. Lovric . Berlin: Springer. 10.1007/978-3-642-04898-2_110.

[eap70314-bib-0002] Albert, D. A. , A. W. Douglas , J. W. Ingram , and T. A. Thompson . 2005. “Hydrogeomorphic Classification for Great Lakes Coastal Wetlands.” Journal of Great Lakes Research 31: 129–146. 10.1016/S0380-1330(05)70294-X.

[eap70314-bib-0003] Anderson, O. , A. Harrison , B. Heumann , C. Godwin , and D. Uzarski . 2023. “The Influence of Extreme Water Levels on Coastal Wetland Extent across the Laurentian Great Lakes.” Science of the Total Environment 885: 163755. 10.1016/j.scitotenv.2023.163755. 37127153

[eap70314-bib-0004] Aplet, G. H. , and P. S. McKinley . 2017. “A Portfolio Approach to Managing Ecological Risks of Global Change.” Ecosystem Health and Sustainability 3(2): e01261. 10.1002/ehs2.1261.

[eap70314-bib-0005] Atamas, N. S. , and O. V. Tomchenko . 2020. “Black Tern Nest‐Site Fidelity in Unstable Habitat: Preliminary Study.” Zoodiversity 54(4): 341–348. 10.15407/zoo2020.04.341.

[eap70314-bib-0006] Bansal, S. , S. Lishawa , B. Tangen , D. Wilcox , D. Albert , M. J. Anteau , M. J. Chimney , R. L. Cressey , E. DeKeyser , and K. J. Elgersma . 2019. “ *Typha* (Cattail) Invasion in North American Wetlands: Biology, Regional Problems, Impacts, Ecosystem Services, and Management.” Wetlands 39(4): 1–40. 10.1007/s13157-019-01174-7.

[eap70314-bib-0007] Barbier, E. B. , S. D. Hacker , C. Kennedy , E. W. Koch , A. C. Stier , and B. R. Silliman . 2011. “The Value of Estuarine and Coastal Ecosystem Services.” Ecological Monographs 81(2): 169–193. 10.1890/10-1510.1.

[eap70314-bib-0008] Bartoń, K. 2022. “MuMIn: Multi‐Model Inference.” R Package Version 1.46.0.” https://CRAN.R-project.org/package=MuMIn.

[eap70314-bib-0009] Bates, D. , M. Maechler , B. Bolker , and S. Walker . 2015. “Fitting Linear Mixed‐Effects Models Using lme4.” Journal of Statistical Software 67: 1–48. 10.18637/jss.v067.i01.

[eap70314-bib-0010] Beisner, B. , D. Haydon , and K. Cuddington . 2003. “Alternative Stable States in Ecology.” Frontiers in Ecology and the Environment 1(7): 376–382. 10.1890/1540-9295(2003)001[0376:ASSIE]2.0.CO;2.

[eap70314-bib-0011] Bergman, R. D. , P. Swain , and M. W. Weller . 1970. “A Comparative Study of Nesting Forster's and Black Terns.” The Wilson Bulletin 82(4): 435–444.

[eap70314-bib-0012] Bielke, S. , J. Grand , I. Grobelna , L. Hardaway , J. Hill‐Gabriel , A. Hinickle , R. Johnson , et al. 2021. Audubon's Vision: Restoring the Great Lakes for Birds and People. New York, NY: National Audubon Society.

[eap70314-bib-0013] Bolsenga, S. J. , and C. E. Herdendorf . 1993. Lake Erie and Lake St. Clair Handbook. Detroit, MI: Wayne State University Press.

[eap70314-bib-0014] Bourgeau‐Chavez, L. , K. Kowalski , M. Mazur , K. Scarbrough , R. Powell , C. Brooks , B. Huberty , et al. 2013. “Mapping Invasive *Phragmites Australis* in the Coastal Great Lakes with ALOS PALSAR Satellite Imagery for Decision Support.” Journal of Great Lakes Research 39: 65–77. 10.1016/j.jglr.2012.11.001.

[eap70314-bib-0015] Bourgeau‐Chavez, L. , S. Endres , M. Battaglia , M. E. Miller , E. Banda , Z. Laubach , P. Higman , P. Chow‐Fraser , and J. Marcaccio . 2015. “Development of a Bi‐National Great Lakes Coastal Wetland and Land Use Map Using Three‐Season PALSAR and Landsat Imagery.” Remote Sensing 7(7): 8655–8682. 10.3390/rs70708655.

[eap70314-bib-0016] Bourgeau‐Chavez, L. L. , K. P. Kowalski , M. J. Battaglia , and A. F. Poley . 2019. “Land cover map including wetlands and invasive Phragmites circa 2017.” U.S. Geological Survey Data Release 10.5066/P9OX21T6.

[eap70314-bib-0017] Bracey, A. , J. Bednar , S. Kolbe , and A. Grinde . 2024. Conserving Black Terns and Forster's Terns in Minnesota, NNRI/TR‐2024/25 Technical Report. Duluth, MN: Natural Resources Research Institute, University of Minnesota Duluth.

[eap70314-bib-0018] Brazner, J. , M. E. Sierzen , J. Keough , and D. Tanner . 2001. “Assessing the Ecological Importance of Coastal Wetlands in a Large Lake Context.” Verhandlungen Des Internationalen Verein Limnologie 27(4): 1950–1961. 10.1080/03680770.1998.11901583.

[eap70314-bib-0019] Carson, B. D. , S. C. Lishawa , N. C. Tuchman , A. M. Monks , B. A. Lawrence , and D. A. Albert . 2018. “Harvesting Invasive Plants to Reduce Nutrient Loads and Produce Bioenergy: An Assessment of Great Lakes Coastal Wetlands.” Ecosphere 9(6): e02320. 10.1002/ecs2.2320.

[eap70314-bib-0020] Cheskey, E. D. , and W. G. Wilson . 2001. *Eastern Lake St. Clair Important Bird Area Conservation Plan*. Can. Nature Fed., Bird Studies Can., Fed. of Ont. Naturalists. 50 pp.

[eap70314-bib-0021] Clites, A. H. , J. P. Smith , T. S. Hunter , and A. D. Gronewold . 2014. “Visualizing Relationships between Hydrology, Climate, and Water Level Fluctuations on Earth's Largest System of Lakes.” Journal of Great Lakes Research 40(3): 807–811. 10.1016/j.jglr.2014.05.014.

[eap70314-bib-0022] Convention on Wetlands . 2021. Global Wetland Outlook: Special Edition 2021. Gland, Switzerland: Secretariat of the Convention on Wetlands.

[eap70314-bib-0023] Côté, I. M. , and E. S. Darling . 2010. “Rethinking Ecosystem Resilience in the Face of Climate Change.” PLoS Biology 8(7): e1000438. 10.1371/journal.pbio.1000438. PMC2910654 20668536

[eap70314-bib-0024] Côté, I. M. , E. S. Darling , and C. J. Brown . 2016. “Interactions among Ecosystem Stressors and their Importance in Conservation.” Proceedings of the Royal Society B: Biological Sciences 283(1824): 20152592. 10.1098/rspb.2015.2592. PMC4760168 26865306

[eap70314-bib-0025] Craig, R. J. , and K. G. Beal . 1992. “The Influence of Habitat Variables on Marsh Bird Communities of the Connecticut River Estuary.” The Wilson Bulletin 104: 295–311.

[eap70314-bib-0026] Crowell, M. , S. Edelman , K. Coulton , and S. McAfee . 2007. “How Many People Live in Coastal Areas?” Journal of Coastal Research 23(5): iii–vi. 10.2112/07A-0017.1.

[eap70314-bib-0027] Cuthbert, N. L. 1954. “A Nesting Study of the Black Tern in Michigan.” The Auk 71(1): 36–63. 10.2307/4081515.

[eap70314-bib-0028] Cuthbert, F. J. , and L. Wires . 2013. The Fourth Decadal U.S. Great Lakes Colonial Waterbird Survey (2007‐2010): Results and Recommendations to Improve the Scientific Basis for Conservation and Management. Minneapolis, MN: Dept. Fisheries, Wildlife & Conservation Biology, University of Minnesota ‐ Twin Cities.

[eap70314-bib-0029] Danz, N. P. , G. J. Niemi , R. R. egal , T. Hollenhorst , L. B. Johnson , J. M. Hanowski , R. P. Axler , et al. 2007. “Integrated Measures of Anthropogenic Stress in the U.S. Great Lakes Basin.” Environmental Management 39(5): 631–647. 10.1007/s00267-005-0293-0. 17387547

[eap70314-bib-0030] Davidson, N. C. 2018. “Wetland Losses and the Status of Wetland‐Dependent Species.” In The Wetland Book: II: Distribution, Description, and Conservation, edited by C. M. Finlayson , G. R. Milton , R. C. Prentice , and N. C. Davidson . Netherlands: Springer. 10.1007/978-94-007-4001-3_197.

[eap70314-bib-0031] Davis, K. L. , S. P. Saunders , S. Beilke , E. R. Ford , J. Fuller , A. Landgraf , and E. F. Zipkin . 2023. “Breeding Season Management Is Unlikely to Improve Population Viability of a Data‐Deficient Migratory Species in Decline.” Biological Conservation 283: 110104. 10.1016/j.biocon.2023.110104.

[eap70314-bib-0032] Derosier, A. L. , S. K. Hanshue , K. E. Wehrly , J. K. Farkas , and M. J. Nichols . 2015. Michigan's Wildlife Action Plan: Great Lakes Marsh and Emergent Wetlands. Lansing, MI: Michigan Department of Natural Resources.

[eap70314-bib-0033] Desgranges, J.‐L. , J. Ingram , B. Drolet , J. Morin , C. Savage , and D. Borcard . 2006. “Modelling Wetland Bird Response to Water Level Changes in the Lake Ontario ‐ St. Lawrence River Hydrosystem.” Environmental Monitoring and Assessment 113(1): 329–365. 10.1007/s10661-005-9087-3. 16518674

[eap70314-bib-0034] Dugan, J. E. , L. Airoldi , M. G. Chapman , S. J. Walker , and T. Schlacher . 2011. “Estuarine and Coastal Structures: Environmental Effects, a Focus on Shore and Nearshore Structures.” In Treatise on Estuarine and Coastal Science, edited by E. Wolanski and D. McLusky , 17–41. Waltham: Academic Press. 10.1016/B978-0-12-374711-2.00802-0.

[eap70314-bib-0035] Fergen, J. T. , R. D. Bergstrom , M. R. Twiss , L. Johnson , A. D. Steinman , and V. Gagnon . 2022. “Updated Census in the Laurentian Great Lakes Watershed: A Framework for Determining the Relationship between the Population and this Aquatic Resource.” Journal of Great Lakes Research 48(6): 1337–1344. 10.1016/j.jglr.2022.03.004.

[eap70314-bib-0036] Firstencel, H. 1987. “The Black Tern (Chlidonias niger Linn.): Breeding Ecology in Upstate New York and Results of Pesticide Residue Analyses.” SUNY Brockport. https://api.semanticscholar.org/CorpusID:131336488.

[eap70314-bib-0037] Folke, C. , S. Carpenter , B. Walker , M. Scheffer , T. Elmqvist , L. Gunderson , and C. S. Holling . 2004. “Regime Shifts, Resilience, and Biodiversity in Ecosystem Management.” Annual Review of Ecology, Evolution, and Systematics 35(1): 557–581. 10.1146/annurev.ecolsys.35.021103.105711.

[eap70314-bib-0038] Fuller, J. , E. Ford , A. Landgraf , K. Alofs , J. Foufopoulos , and A. Gronewold . 2026. “Dataset for ‘Long‐term monitoring and remote sensing reveal waterbird colony collapses with rising lake‐levels and multiple stressors’.” Zenodo. [Dataset] 10.5281/zenodo.19290242 PMC13620196 42806475

[eap70314-bib-0039] Gao, C. , K.‐H. Knorr , H. Chen , and Y. He . 2023. “Editorial: Disturbance, Resilience, and Restoration of Wetlands.” Frontiers in Ecology and Evolution 11: 1166223. 10.3389/fevo.2023.1166223.

[eap70314-bib-0040] Gardner, R. C. , and C. Finlayson . 2018. Global Wetland Outlook: State of the World's Wetlands and their Services to People, Research Paper Nos. 2020–5. Gland, Switzerland: Ramsar Convention Secretariat https://papers.ssrn.com/abstract=3261606.

[eap70314-bib-0041] Gerson, H. 1988. Status Report on the Black Tern (Chlidonias Niger). Toronto, Ontario: Committee on the Status of Endangered Wildlife in Canada (COSEWIC).

[eap70314-bib-0042] Gilbert, A. T. , and F. A. Servello . 2005. “Water Level Dynamics in Wetlands and Nesting Success of Black Terns in Maine.” Waterbirds 28(2): 181–187. 10.1675/1524-4695(2005)028[0181:WLDIWA]2.0.CO;2.

[eap70314-bib-0043] Gnass Giese, E. E. , R. W. Howe , A. T. Wolf , and G. J. Niemi . 2018. “Breeding Birds and Anurans of Dynamic Coastal Wetlands in Green Bay, Lake Michigan.” Journal of Great Lakes Research 44(5): 950–959. 10.1016/j.jglr.2018.06.003.

[eap70314-bib-0044] Golawski, A. , and E. Mroz . 2019. “Differences in Nest Site Characteristics and Hatching Success in White‐Winged Tern (*Chlidonias leucopterus*) and Black Tern (*Chlidonias niger*).” Hydrobiologia 828(1): 1–10. 10.1007/s10750-018-3791-9.

[eap70314-bib-0045] Grand, J. , S. Saunders , N. Michel , L. Elliott , S. Beilke , A. Bracey , T. Gehring , et al. 2020. “Prioritizing Coastal Wetlands for Marsh Bird Conservation in the U.S. Great Lakes.” Biological Conservation 249: 108708. 10.1016/j.biocon.2020.108708.

[eap70314-bib-0046] Gronewold, A. D. , J. Bruxer , D. Durnford , J. P. Smith , A. H. Clites , F. Seglenieks , S. S. Qian , T. S. Hunter , and V. Fortin . 2016. “Hydrological Drivers of Record‐Setting Water Level Rise on Earth's Largest Lake System.” Water Resources Research 52(5): 4026–4042. 10.1002/2015WR018209.

[eap70314-bib-0047] Gronewold, A. D. , H. X. Do , Y. Mei , and C. A. Stow . 2021. “A Tug‐of‐War within the Hydrologic Cycle of a Continental Freshwater Basin.” Geophysical Research Letters 48(4): e2020GL090374. 10.1029/2020GL090374.

[eap70314-bib-0048] Hanski, I. , and D. Simberloff . 1997. “The Metapopulation Approach, its History, Conceptual Domain, and Application to Conservation.” In Metapopulation Biology, edited by I. Hanski and M. E. Gilpin , 5–26. San Diego: Academic Press.

[eap70314-bib-0049] Hays, H. , and M. Lecroy . 1971. “Field Criteria for Determining Incubation Stage in Eggs of the Common Tern.” The Wilson Bulletin 83(4): 425–429.

[eap70314-bib-0050] Heath, S. R. 2004. Nest Success and Chick Survival of Black Terns in Maine: Effects of Predation on Breeding Productivity. Orono, ME: The University of Maine https://digitalcommons.library.umaine.edu/etd/642.

[eap70314-bib-0051] Heath, S. R. , E. H. Dunn , and D. J. Agro . 2020. “Black Tern (Chlidonias Niger), Version 1.0.” In Birds of the World, edited by S. M. Billerman . Ithaca, NY: Cornell Lab of Ornithology. 10.2173/bow.blkter.01.

[eap70314-bib-0052] Hennen, M. J. , and M. L. Brown . 2014. “Movement and Spatial Distribution of Common Carp in a South Dakota Glacial Lake System: Implications for Management and Removal.” North American Journal of Fisheries Management 34(6): 1270–1281. 10.1080/02755947.2014.959674.

[eap70314-bib-0053] Hickey, J. M. , and R. A. Malecki . 1997. “Nest Site Selection of the Black Tern in Western New York.” Colonial Waterbirds 20: 582. 10.2307/1521614.

[eap70314-bib-0054] Hogue, A. S. , and K. Breon . 2022. “The Greatest Threats to Species.” Conservation Science and Practice 4(5): e12670. 10.1111/csp2.12670.

[eap70314-bib-0055] Isbell, F. , P. Balvanera , A. S. Mori , J. S. He , J. M. Bullock , G. R. Regmi , E. W. Seabloom , et al. 2023. “Expert Perspectives on Global Biodiversity Loss and its Drivers and Impacts on People.” Frontiers in Ecology and the Environment 21(2): 94–103. 10.1002/fee.2536.

[eap70314-bib-0056] Jacob, L. M. , K. N. Irvine , B. B. Beza , and L. H. C. Chua . 2025. “Adaptive Resilience in Wetlands: An Integrative Review of Principles, Research Gaps, and Ways Forward for Better Adaptive Management.” Ecological Engineering 220: 107720. 10.1016/j.ecoleng.2025.107720.

[eap70314-bib-0057] Johst, K. , R. Brandl , and S. Eber . 2002. “Metapopulation Persistence in Dynamic Landscapes: The Role of Dispersal Distance.” Oikos 98(2): 263–270. 10.1034/j.1600-0706.2002.980208.x.

[eap70314-bib-0058] Kantrud, H. A. , and R. E. Stewart . 1984. “Ecological Distribution and Crude Density of Breeding Birds on Prairie Wetlands.” The Journal of Wildlife Management 48(2): 426. 10.2307/3801174.

[eap70314-bib-0059] Krieger, K. A. 1992. “The Ecology of Invertebrates in Great Lakes Coastal Wetlands: Current Knowledge and Research Needs.” Journal of Great Lakes Research 18(4): 634–650. 10.1016/S0380-1330(92)71327-6.

[eap70314-bib-0060] Lougheed, V. , B. Crosbie , and P. Chow‐Fraser . 1998. “Predictions on the Effect of Common Carp (*Cyprinus Carpio*) Exclusion on Water Quality, Zooplankton, and Submergent Macrophytes in a Great Lakes Wetland.” Canadian Journal of Fisheries and Aquatic Sciences 55(5): 1189–1197. 10.1139/cjfas-55-5-1189.

[eap70314-bib-0061] Matteson, S. W. , M. J. Mossman , and D. A. Shealer . 2012. “Population Decline of Black Terns in Wisconsin: A 30‐Year Perspective.” Waterbirds 35(2): 185–193. 10.1675/063.035.0201.

[eap70314-bib-0062] Matthiopoulos, J. , J. Harwood , and L. Thomas . 2005. “Metapopulation Consequences of Site Fidelity for Colonially Breeding Mammals and Birds.” Journal of Animal Ecology 74(4): 716–727. 10.1111/j.1365-2656.2005.00970.x.

[eap70314-bib-0063] Maxson, S. J. , J. R. Fieberg , and M. R. Riggs . 2007. “Black Tern Nest Habitat Selection and Factors Affecting Nest Success in Northwestern Minnesota.” Waterbirds 30(1): 1–9. https://www.jstor.org/stable/4501788.

[eap70314-bib-0064] McFeeters, S. K. 1996. “The Use of the Normalized Difference Water Index (NDWI) in the Delineation of Open Water Features.” International Journal of Remote Sensing 17(7): 1425–1432. 10.1080/01431169608948714.

[eap70314-bib-0065] Meli, P. , J. M. Rey Benayas , P. Balvanera , and M. Martínez Ramos . 2014. “Restoration Enhances Wetland Biodiversity and Ecosystem Service Supply, but Results Are Context‐Dependent: A Meta‐Analysis.” PLoS One 9(4): e93507. 10.1371/journal.pone.0093507. PMC3990551 24743348

[eap70314-bib-0066] Millennium Ecosystem Assessment . 2005. Ecosystems and Human Well‐Being: Wetlands and Water Synthesis: A Report of the Millennium Ecosystem Assessment. Washington, DC: World Resources Institute.

[eap70314-bib-0067] Mitsch, W. 2016. “Wetlands and Climate Change.” National Wetlands Newsletter 38(1): 5–11.

[eap70314-bib-0068] Mortsch, L. , J. Ingram , A. Hebb , and S. Doka . 2006. Great Lakes Coastal Wetland Communities: Vulnerability to Climate Change and Response to Adaptation Strategies. Ottawa, Ontario: Climate Change Impacts and Adaptation Program, Natural Resources Canada.

[eap70314-bib-0069] Mosher, B. A. 1986. Factors Influencing Reproductive Success and Nesting Strategies in Black Terns. Burnaby, British Columbia: Simon Fraser University.

[eap70314-bib-0070] Moy, M. 1995. “Alarm Calls and Mobbing Behavior Associated with Predation Risk in Nesting Black Terns (*Chlidonias niger*).” Working Paper. Ann Arbor, MI: Biological Station, University of Michigan (UMBS). http://deepblue.lib.umich.edu/handle/2027.42/54619.

[eap70314-bib-0071] Naugle, D. E. , R. R. Johnson , M. E. Estey , and K. F. Higgins . 2000. “A Landscape Approach to Conserving Wetland Bird Habitat in the Prairie Pothole Region of Eastern South Dakota.” Wetlands 20(1): 588–604. 10.1672/0277-5212(2000)020[0588:ALATCW]2.0.CO;2.

[eap70314-bib-0072] Orr, J. A. , R. D. Vinebrooke , M. C. Jackson , K. J. Kroeker , R. L. Kordas , C. Mantyka‐Pringle , P. J. Van den Brink , et al. 2020. “Towards a Unified Study of Multiple Stressors: Divisions and Common Goals across Research Disciplines.” Proceedings of the Royal Society B: Biological Sciences 287(1926): 20200421. 10.1098/rspb.2020.0421. PMC7282922 32370677

[eap70314-bib-0073] Pardieck, K. L. , D. Ziolkowski, Jr. , M. Lutmerding , V. Aponte , and M.‐A. R. Hudson . 2020. “North American Breeding Bird Survey Dataset 1966–2019, Version 2019.0.” U.S. Geological Survey Data Release. 10.5066/P9J6QUF6.

[eap70314-bib-0074] Perrins, C. M. 1970. “The Timing of Birds' Breeding Seasons.” Ibis 112(2): 242–255. 10.1111/j.1474-919X.1970.tb00096.x.

[eap70314-bib-0075] Peterson, S. H. , J. T. Ackerman , M. P. Keating , C. R. Schacter , C. A. Hartman , M. L. Casazza , and M. P. Herzog . 2022. “Predator Movements in Relation to Habitat Features Reveal Vulnerability of Duck Nests to Predation.” Ecology and Evolution 12(9): e9329. 10.1002/ece3.9329. PMC9487884 36188493

[eap70314-bib-0076] Pirotta, E. , L. Thomas , D. P. Costa , A. J. Hall , C. M. Harris , J. Harwood , S. D. Kraus , et al. 2022. “Understanding the Combined Effects of Multiple Stressors: A New Perspective on a Longstanding Challenge.” Science of the Total Environment 821: 153322. 10.1016/j.scitotenv.2022.153322. 35074373

[eap70314-bib-0077] Pontee, N. 2013. “Defining Coastal Squeeze: A Discussion.” Ocean & Coastal Management 84: 204–207. 10.1016/j.ocecoaman.2013.07.010.

[eap70314-bib-0078] Prince, H. H. , P. I. Padding , and R. W. Knapton . 1992. “Waterfowl Use of the Laurentian Great Lakes.” Journal of Great Lakes Research 18(4): 673–699. 10.1016/S0380-1330(92)71329-X.

[eap70314-bib-0079] Quinn, F. H. , and C. E. Sellinger . 1990. “Lake Michigan Record Levels of 1838, a Present Perspective.” Journal of Great Lakes Research 16(1): 133–138. 10.1016/S0380-1330(90)71405-0.

[eap70314-bib-0080] R Core Team . 2025. R: A Language and Environment for Statistical Computing. Vienna, Austria: R Foundation for Statistical Computing. http://www.R-project.org/.

[eap70314-bib-0081] Reid, M. A. , S. Chilcott , and M. C. Thoms . 2018. “Using Palaeoecological Records to Disentangle the Effects of Multiple Stressors on Floodplain Wetlands.” Journal of Paleolimnology 60(2): 247–271. 10.1007/s10933-017-0011-y.

[eap70314-bib-0082] Robichaud, C. D. , and R. C. Rooney . 2017. “Long‐Term Effects of a *Phragmites australis* Invasion on Birds in a Lake Erie Coastal Marsh.” Journal of Great Lakes Research 43(3): 141–149. 10.1016/j.jglr.2017.03.018.

[eap70314-bib-0083] Robin, X. , N. Turck , A. Hainard , N. Tiberti , F. Lisacek , J.‐C. Sanchez , and M. Müller . 2011. “pROC: An Open‐Source Package for R and S+ to Analyze and Compare ROC Curves.” BMC Bioinformatics 12: 77. 10.1186/1471-2105-12-77. PMC3068975 21414208

[eap70314-bib-0084] Roth, R. , D. Hart , R. Mead , and C. Quinn . 2016. “Wireframing for Interactive & web‐Based Geographic Visualization: Designing the NOAA Lake Level Viewer.” Cartography and Geographic Information Science 44(4): 338–357. 10.1080/15230406.2016.1171166.

[eap70314-bib-0085] Sauer, J. , J. E. Hines , J. E. Fallon , K. L. Pardieck , D. J. Ziolkowski, Jr. , and W. A. Link . 2014. “The North American Breeding Bird Survey, Results and Analysis 1966–2012.” Version 02.19.2014. Laurel, MD: USGS Patuxent Wildlife Research Center.

[eap70314-bib-0086] Sauer, J. R. , D. K. Niven , J. E. Hines , D. J. Ziolkowski, Jr. , K. L. Pardieck , J. E. Fallon , and W. A. Link . 2019. The North American Breeding Bird Survey, Results and Analysis 1966–2019. Version 2.07.2019. Laurel, MD: USGS Patuxent Wildlife Research Center.

[eap70314-bib-0087] Saunders, S. P. , K. A. L. Hall , N. Hill , and N. L. Michel . 2019. “Multiscale Effects of Wetland Availability and Matrix Composition on Wetland Breeding Birds in Minnesota, USA.” The Condor 121(3): duz024. 10.1093/condor/duz024.

[eap70314-bib-0088] Shealer, D. A. , J. M. Buzzell , and J. P. Heiar . 2006. “Effect of Floating Nest Platforms on the Breeding Performance of Black Terns.” Journal of Field Ornithology 77: 184–194. 10.1111/j.1557-9263.2006.00040.x.

[eap70314-bib-0089] Shephard, N. G. , P. Szczys , D. J. Moore , M. W. Reudink , J. N. Costa , A. M. Bracey , S. Lisovski , and A. E. McKellar . 2023. “Weak Genetic Structure, Shared Nonbreeding Areas, and Extensive Movement in a Declining Waterbird.” Ornithological Applications 125(1): duac053. 10.1093/ornithapp/duac053.

[eap70314-bib-0090] Signorell, A. 2025. “DescTools: Tools for Descriptive Statistics.” R package version 0.99.60. https://CRAN.R-project.org/package=DescTools.

[eap70314-bib-0091] Soulliere, G. J. , M. A. Al‐Saffar , R. L. Pierce , M. J. Monfils , L. R. Wires , B. W. Loges , B. T. Shirkey , et al. 2018. Upper Mississippi River and Great Lakes Region Joint Venture Waterbird Habitat Conservation Strategy – 2018 Revision. Bloomington, MN: U.S. Fish and Wildlife Service.

[eap70314-bib-0092] Staudt, A. , A. K. Leidner , J. Howard , K. A. Brauman , J. S. Dukes , L. J. Hansen , C. Paukert , J. Sabo , and L. A. Solórzano . 2013. “The Added Complications of Climate Change: Understanding and Managing Biodiversity and Ecosystems.” Frontiers in Ecology and the Environment 11(9): 494–501. 10.1890/120275.

[eap70314-bib-0093] Steen, V. A. , and A. N. Powell . 2012. “Wetland Selection by Breeding and Foraging Black Terns in the Prairie Pothole Region of the United States.” The Condor: Ornithological Applications 114(1): 155–165. 10.1525/cond.2012.110097.

[eap70314-bib-0094] Swearingen, J. , K. Saltonstall , and D. Tilley . 2012. Phragmites Field Guide: Distinguishing Native and Exotic Forms of Common Reed (Phragmites Australis) in the United States. USDA‐NRCS Plant Materials Technical Note No. 56. Boise, ID: U.S. Department of Agriculture, Natural Resources Conservation Service.

[eap70314-bib-0095] Timmermans, S. T. A. , S. S. Badzinski , and J. W. Ingram . 2008. “Associations between Breeding Marsh Bird Abundances and Great Lakes Hydrology.” Journal of Great Lakes Research 34(2): 351–364. 10.3394/0380-1330(2008)34[351:ABBMBA]2.0.CO;2.

[eap70314-bib-0096] Tozer, D. 2015. “Marsh Bird Occupancy Dynamics, Trends, and Conservation in the Southern Great Lakes Basin: 1996 to 2013.” Journal of Great Lakes Research 42(1): 136–145. 10.1016/j.jglr.2015.10.015.

[eap70314-bib-0097] Tozer, D. C. 2020. Great Lakes Marsh Monitoring Program: 25 Years of Conserving Birds and Frogs. Port Rowan, Ontario: Birds Canada.

[eap70314-bib-0098] Tozer, D. C. , A. M. Bracey , G. E. Fiorino , T. M. Gehring , E. E. Gnass Giese , G. P. Grabas , R. W. Howe , et al. 2024. “Increasing Marsh Bird Abundance in Coastal Wetlands of the Great Lakes, 2011‐2021, Likely Caused by Increasing Water Levels.” Ornithological Applications 126(2): duad062.

[eap70314-bib-0099] Trebitz, A. , M. Sykes , and J. Barge . 2019. “A Reference Inventory for Aquatic Fauna of the Laurentian Great Lakes.” Journal of Great Lakes Research 45(6): 1036–1046. 10.1016/j.jglr.2019.10.004. PMC8318194 34326568

[eap70314-bib-0100] Tucker, C. J. , and P. J. Sellers . 1986. “Satellite Remote Sensing of Primary Production.” International Journal of Remote Sensing 7(11): 1395–1416. 10.1080/01431168608948944.

[eap70314-bib-0101] Tulbure, M. G. , and C. A. Johnston . 2010. “Environmental Conditions Promoting Non‐Native *Phragmites australis* Expansion in Great Lakes Coastal Wetlands.” Wetlands 30(3): 577–587. 10.1007/s13157-010-0054-6.

[eap70314-bib-0102] Tulbure, M. G. , C. A. Johnston , and D. L. Auger . 2007. “Rapid Invasion of a Great Lakes Coastal Wetland by Non‐Native *Phragmites australis* and *Typha* .” Journal of Great Lakes Research 33(3): 269–279. 10.3394/0380-1330(2007)33[269:RIOAGL]2.0.CO;2.

[eap70314-bib-0103] USGCRP . 2018. Second State of the Carbon Cycle Report. Washington, DC: U.S. Global Change Research Program https://carbon2018.globalchange.govhttps://carbon2018.globalchange.gov/chapter/executive-summary.

[eap70314-bib-0104] Uzarski, D. G. , V. J. Brady , M. J. Cooper , D. A. Wilcox , D. A. Albert , R. P. Axler , P. Bostwick , et al. 2017. “Standardized Measures of Coastal Wetland Condition: Implementation at a Laurentian Great Lakes Basin‐Wide Scale.” Wetlands 37: 15–32. 10.1007/s13157-016-0835-7.

[eap70314-bib-0105] VanDeWeghe, A. , V. Lin , J. Jayaram , and A. D. Gronewold . 2022. “Changes in Large Lake Water Level Dynamics in Response to Climate Change.” Frontiers in Water 4: 805143. 10.3389/frwa.2022.805143.

[eap70314-bib-0106] Verhulst, S. , and J.‐Å. Nilsson . 2008. “The Timing of Birds' Breeding Seasons: A Review of Experiments that Manipulated Timing of Breeding.” Philosophical Transactions of the Royal Society B: Biological Sciences 363(1490): 399–410. 10.1098/rstb.2007.2146. PMC2606757 17666390

[eap70314-bib-0107] Walker, B. , C. S. Holling , S. R. Carpenter , and A. Kinzig . 2004. “Resilience, Adaptability and Transformability in Social‐Ecological Systems.” Ecology and Society 9(2): art5. 10.5751/ES-00650-090205.

[eap70314-bib-0108] Weier, J. , and P. J. Sellers . 2000. “Measuring Vegetation (NDVI & EVI).” NASA Earth Observatory. https://earthobservatory.nasa.gov/features/MeasuringVegetation.

[eap70314-bib-0109] Weller, M. W. , and C. S. Spatcher . 1965. Role of Habitat in the Distribution and Abundance of Marsh Birds. Ames, Iowa: Agricultural and Home Economics Experiment Station, Iowa State University of Science and Technology.

[eap70314-bib-0110] Wensink, S. , and S. Tiegs . 2016. “Shoreline Hardening Alters Freshwater Shoreline Ecosystems.” Freshwater Science 35(3): 764–777. 10.1086/687279.

[eap70314-bib-0111] Wilcox, D. A. 2004. “Implications of Hydrologic Variability on the Succession of Plants in Great Lakes Wetlands.” Aquatic Ecosystem Health & Management 7(2): 223–231. 10.1080/14634980490461579.

[eap70314-bib-0112] Wilcox, D. A. 2012. “Response of Wetland Vegetation to the Post‐1986 Decrease in Lake St. Clair Water Levels: Seed‐Bank Emergence and Beginnings of the *Phragmites australis* Invasion.” Journal of Great Lakes Research 38(2): 270–277. 10.1016/j.jglr.2012.02.007.

[eap70314-bib-0113] Wilcox, D. A. , K. P. Kowalski , H. L. Hoare , M. L. Carlson , and H. N. Morgan . 2008. “Cattail Invasion of Sedge/Grass Meadows in Lake Ontario: Photointerpretation Analysis of Sixteen Wetlands over Five Decades.” Journal of Great Lakes Research 34(2): 301–323. 10.3394/0380-1330(2008)34[301:CIOGMI]2.0.CO;2.

[eap70314-bib-0114] Wu, H. , A. J. R. Godfrey , K. Govindaraju , and S. Pirikahu . 2025. “ExtDist: Extending the Range of Functions for Probability Distributions.” R Package Version 0.74. https://CRAN.R-project.org/package=ExtDist.

[eap70314-bib-0115] Wyman, K. E. , and F. J. Cuthbert . 2017. “Black Tern (*Chlidonias niger*) Breeding Site Abandonment in U.S. Great Lakes Coastal Wetlands Is Predicted by Historical Abundance and Patterns of Emergent Vegetation.” Wetlands Ecology and Management 25(5): 583–596. 10.1007/s11273-017-9537-z.

[eap70314-bib-0116] Zimmerman, A. L. , J. A. Dechant , B. E. Johnson , and B. R. Euliss . 2002. “Effects of Management Practices on Wetland Birds: Black Tern. Northern Prairie Wildlife Research Center, Jamestown, ND.” Jamestown, ND: Northern Prairie Wildlife Research Center. 10.3133/70159831.

