# Supplementary material for: Long‐term monitoring and remote sensing reveal waterbird colony collapses with rising lake levels and multiple stressors
Source: Ecol Appl. 2026 Sep 28;36(6):e70314. doi: 10.1002/eap.70314 (PMC13620196; doi:10.1002/eap.70314)
Supplement: Supplementary file 1 [file EAP-36-e70314-s001.pdf]

Appendix S8

Long-term monitoring and remote sensing reveal waterbird colony collapses with rising lake levels and multiple stressors

Jennifer L. Fuller, Erin Ford, Ava Landgraf, Johannes Foufopoulos, Andrew Gronewold, Karen M. Alofs

Ecological Applications

Appendix S8. GLM Map Model Application

Using the focal statistics tool, each year's NDVI layer was converted so that every grid cell represented the average surrounding NDVI within a circular 7-m radius. The Euclidean distance tool was used to create a continuous raster where each cell represented distance to the main lake and housing. For percentages of thick vegetation, raster images were first re-classed, so the thick vegetation class was equal to 1, and the surrounding area equal to 0. The "focal statistics" tool in ArcGIS Pro then calculated the sum for each cell within a 7-m radius and was rescaled from 0 to 100 to reflect percentage values.

To visualize changes in the geographic extent of the suitable nesting habitat, we applied the final generalized linear mixed-effects model (GLMM) to raster layers of spatially explicit predictors. To maintain consistency between the GLMM estimation and spatial predictions, we standardized spatial covariates by applying the same means and standard deviations used during model fitting. Because clutch initiation was not spatially explicit, this was held as a constant standardized mean value during spatial prediction. We also incorporated sub-colony as a random effect by extracting their conditional modes (best

linear unbiased predictors) from the final GLMM using the ranef() function from the 'lme4' package in R (Bates et al., 2015). For each year, we calculated the linear predictor of each raster cell by taking the sum of the fixed-effect intercept, the product of the fixed-effects coefficients and standardized raster covariates, and the rasterized sub-colony random intercept. We then calculated the hatching success probabilities for each cell by applying the inverse logit transformation to the linear predictor. In the binomial GLMM, the probability of a nest hatching on a scale of 0 to 1 (p) can be expressed using the equation described in McCullagh & Nelder (2019):

$$p = \frac{1}{1 + e^{-\eta}}$$

Where η represents the linear combination which incorporates the fixed-effect intercept (β_0), fixed-effect slope coefficients (β_k), associated standardized predictors (X_k), and the random intercept (b_s) of the random effect, sub-colony (s):

$$\eta = \beta_0 + \sum_{k=1}^{K} \beta_k X_k + b_s$$

Literature Cited

Bates, D., M. Maechler, B. Bolker, and S. Walker. 2015. “Fitting Linear Mixed-Effects Models Using lme4.” *Journal of Statistical Software* 67: 1-48. doi:10.18637/jss.v067.i01.

McCullagh, P., and J. A. Nelder. 2019. *Generalized Linear Models (2nd ed.).* New York, NY: Routledge.
