# Supplementary material for: Long‐term monitoring and remote sensing reveal waterbird colony collapses with rising lake levels and multiple stressors
Source: Ecol Appl. 2026 Sep 28;36(6):e70314. doi: 10.1002/eap.70314 (PMC13620196; doi:10.1002/eap.70314)
Supplement: Supplementary file 2 [file EAP-36-e70314-s002.pdf]

Appendix S7

Long-term monitoring and remote sensing reveal waterbird colony collapses with rising lake levels and multiple stressors

Jennifer L. Fuller, Erin Ford, Ava Landgraf, Johannes Foufopoulos, Andrew Gronewold, Karen M. Alofs

Ecological Applications

Main Lake Classification

NDWI classes were simplified to reduce open water "pockets" using an ascending boundary clean function, followed by converting the open water class into a multipart polygon. The polygon was exported such that only the main lake polygon was selected, i.e., excluding the inner "pockets" of open water within primarily vegetated areas. Because high resolution imagery could not be obtained in 2015, the 2016 polygon delineation was used. 2016 was chosen as an appropriate representative due to the minimal differences in mean surface lake level between years (0.094 m) during the nesting season (May 15th – July 28th). Similarly, for the "South Channel" or "SCHAN" colony, 2019 lake distances could not be obtained and 2020 was used as the difference between years was also minimal (0.04 m) (Clites et al., 2014).

Literature Cited

Clites, A. H., J. P. Smith, T. S. Hunter, and A. D. Gronewold. 2014. "Visualizing Relationships between Hydrology, Climate, and Water Level Fluctuations on Earth's Largest System of Lakes." *Journal of Great Lakes Research* 40(3): 807–811. https://doi.org/10.1016/j.jglr.2014.05.014.
