# Supplementary material for: Long‐term monitoring and remote sensing reveal waterbird colony collapses with rising lake levels and multiple stressors
Source: Ecol Appl. 2026 Sep 28;36(6):e70314. doi: 10.1002/eap.70314 (PMC13620196; doi:10.1002/eap.70314)
Supplement: Supplementary file 3 [file EAP-36-e70314-s003.pdf]

Appendix S1

Long-term monitoring and remote sensing reveal waterbird colony collapses with rising lake levels and multiple stressors

Jennifer L. Fuller, Erin Ford, Ava Landgraf, Johannes Foufopoulos, Andrew Gronewold, Karen M. Alofs

Ecological Applications

Data Collection and Population Estimates

All nesting data, including journal entries, banding data, and nesting success, is stored with Audubon Great Lakes and Detroit Audubon as hardcopies, images of hardcopies, excel files, and within the Audubon Society's organizational ArcGIS Online account. Findings from each year are additionally compiled into annual reports, which include detailed methodology, results summary, and a discussion with future recommendations. Aside from records becoming more frequent and detailed, nest monitoring methods were kept consistent each year through a lead primary nest monitoring researcher working or volunteering for Detroit Audubon. The lead researcher was responsible for collecting nest data for almost all site visits and training subsequent leads. Between 2013 and 2016, nest monitoring was led by Randall Kling and supported primarily by Caleb Putnam. He trained and transferred his role to Erin Rowan in 2016. Caleb Putnam primarily assisted with Erin Rowan as well until 2018. In 2018, Erin trained Ava Landgraf, who became the primary lead for Detroit Audubon in 2020. Jennifer Fuller primarily assisted with Erin and Ava in 2019 and 2020. Primary nest monitoring researchers were always accompanied by at least one other person (usually the primary research assistant, along

with additional volunteers when they were available) to assist with data collection and ensure field safety. All additional volunteers are credited in the annual reports.

Some nesting data (n = 18) was supplemented by trail cameras in 2018 and 2020 (see **Figure S1** for example of nest destruction by wave action). In 2018, Plotwatcher Pro cameras were set to timelapse and were able to determine 6 out of 8 final outcomes. In 2020, Spypoint Solar Dark and Bushnell Trophy cam E3 cameras were deployed at 1-minute timelapse and determined 10 out of 10 final outcomes. Additional camera data after 2020 captured important information that was observed but not formally documented during the years of the study (e.g., nesting disturbance by spawning carp aggregations; **Figure S2**).

Monitoring for population sizes included flushing sub-colony nesting sites as briefly as possible to avoid too much nest disturbance and counting the number of Black Terns in the area. Headcounts are used to identify how many nests are active and ensure that all nests are checked, eliminating uncertainty of nests with a final unknown status. In addition, they are used for estimating sub-colony and overall population sizes. In most instances, the maximum flushed headcount was used for the total and sub-colony population size as it typically exceeded the number of nests found (prior to any major nest failure events). However, the number of coincident nests (prior to major failure events) were compared to sub-colony counts, as in some rare cases these exceeded the total head count. This can occur if the adults are less prone to flushing (usually closer to the hatch date) or out foraging. If the estimated sub-colony population using nest counts (1 nest = 2

breeding adults) exceeded the flushed head count prior to large failure events, these were used to supplement the sub-colony and overall population size estimate. It should be noted that headcounts are only a “best estimate” and include unavoidable uncertainties due to variable site accessibility, technician error, and the dynamic nature of re-nesting, re-location, and flushing behavior (influenced by individual and/or nesting stage). When monitoring nesting success, colonies that were small and isolated were at times given lower priority and visited less frequently.

Towards the end of the breeding season, head counts of fledglings were useful for confirming the success of a sub-colony. Due to the sheer size of SCF, sighting successful fledglings is difficult and likely an underestimation. However, chicks appear to spend time in their natal sub-colony at least within their first week of fledging. Monitoring fledgling success improved in 2019 after extensive fledgling geo-locator tracking by Alex Jahn from Indiana University. Unfortunately, fledgling success in 2020 was believed to be extremely poor, though highly accurate given the low population size that enabled researchers to confidently identify which sub-colonies and nests failed or succeeded. No geo-locator tracking occurred in 2020 due to low productivity levels, therefore, this could not have contributed to poor fledgling success.

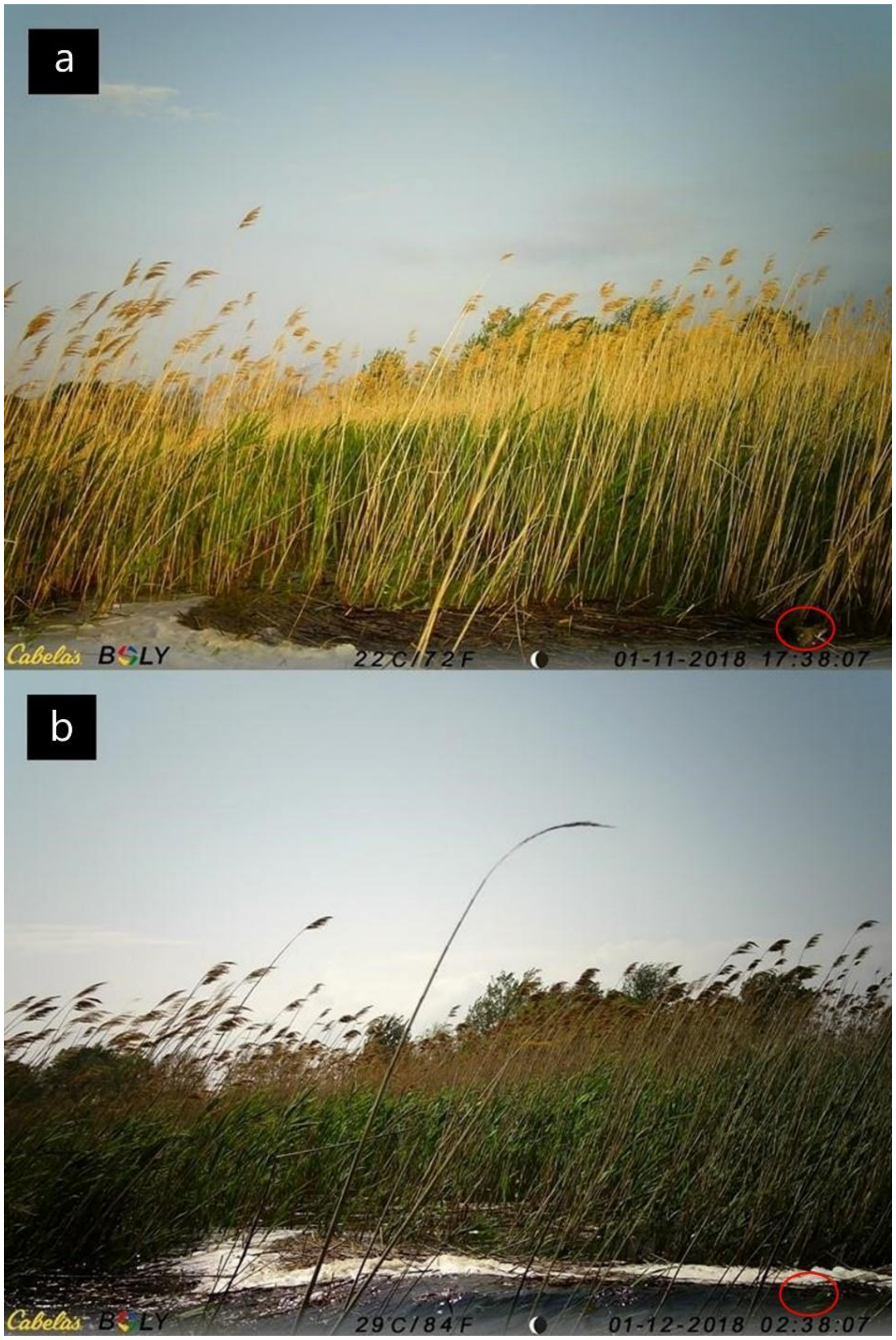

Figure S1. Nest 311 (circled in red) in Little Muscamoot (W) sub-colony on edge of common reed when (A) the adult was last on the nest and (B) a large wave event occurred which destroyed the nest and mat. An error occurred in the camera's timestamp recording and actual time after correction are (A) 7:10 and (B) 16:10 on 6/12/2020. Photo credit: Jennifer Fuller.

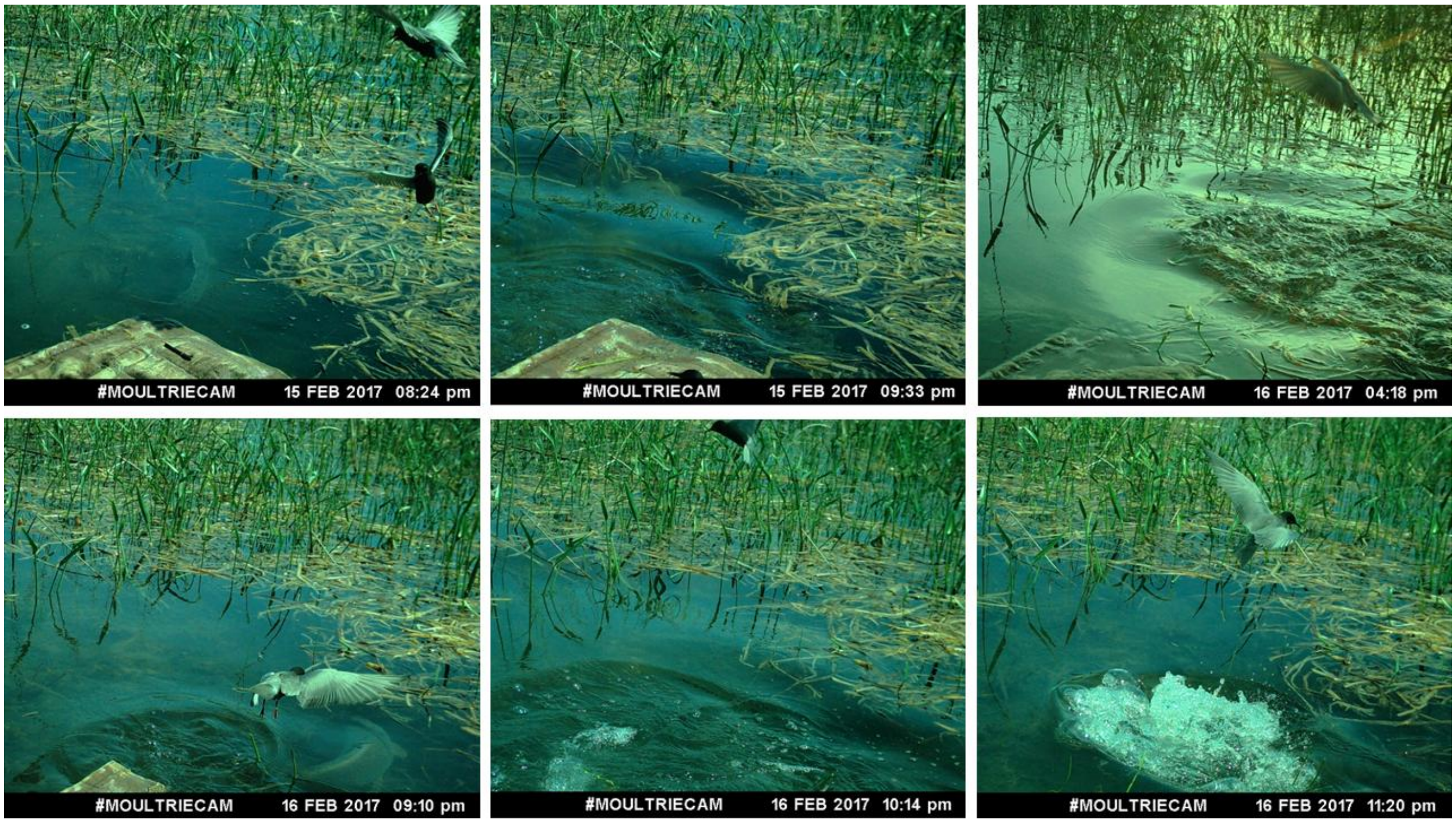

Figure S2. Nest camera data documenting spawning carp disturbing a Black Tern nest on a provided platform in 2025. Listed dates and times in the photos are incorrect due to a camera setting malfunction. Photo credit: Ava Landgraf.
