# Supplementary material for: Long‐term monitoring and remote sensing reveal waterbird colony collapses with rising lake levels and multiple stressors
Source: Ecol Appl. 2026 Sep 28;36(6):e70314. doi: 10.1002/eap.70314 (PMC13620196; doi:10.1002/eap.70314)
Supplement: Supplementary file 4 [file EAP-36-e70314-s004.pdf]

Appendix S5

Long-term monitoring and remote sensing reveal waterbird colony collapses with rising lake levels and multiple stressors

Jennifer L. Fuller, Erin Ford, Ava Landgraf, Johannes Foufopoulos, Andrew Gronewold, Karen M. Alofs

Ecological Applications

Geospatial Variable and Imagery Selection

We chose geospatial habitat variables and sources based on previous literature and the resolutions of available imagery. Black Terns prefer habitats with vegetation percentages ranging between 25-75% (Craig & Beal, 1992; Prince et al., 1992; Wilcox, 2012; Bourgeau-Chavez et al., 2015; Gnass Giese et al., 2018; Heath et al. 2020). However, this does not fully address the true habitat complexity of their breeding grounds. Vegetation type and structure varies across different wetlands used by Black Terns and even within a single colony. In Lake St. Clair, we observed that the largest sub-colonies primarily chose areas that contained plentiful floating, dead plant material, often secured by semi-dispersed cattail or bulrush. Black Terns avoided nesting in densely packed cattail and the highly invasive common reed which impede takeoff and landing, visibility, and prevent mat build-up required for nest construction. We therefore used remote sensing methods to reconstruct vegetation classes corresponding to nesting data collected over previous years. We used ground-truth observations to determine that habitat structure could be generalized into four classes based on stand density: 1) dense, standing vegetation, 2) mat and scattered or cut standing vegetation, 3) sparse standing vegetation and/or sparse, scattered mat, 4)

open water. Another important consideration in terms of nesting habitat is scale. The scale of habitat structure impacting the nest could be very fine, as nests are generally less than 12 inches in diameter (Heath et al., 2020). Previous studies have examined vegetation within a 12-m radius (Hickey & Malecki, 1997) or 2-m radius of nests (Maxson et al., 2007). We selected 3-m, 12-m, and 7-m (median of 2 and 12-m) radii for comparison and chose the radius with the lowest AIC and highest AUC value.

Literature Cited

Bourgeau-Chavez, L., S. Endres, M. Battaglia, M. E. Miller, E. Banda, Z. Laubach, P. Higman, P. Chow-Fraser, and J. Marcaccio. 2015. "Development of a Bi-National Great Lakes Coastal Wetland and Land Use Map Using Three-Season PALSAR and Landsat Imagery." *Remote Sensing* 7(7): 8655–8682. https://doi.org/10.3390/rs70708655.

Craig, R. J., and K. G. Beal. 1992. "The Influence of Habitat Variables on Marsh Bird Communities of the Connecticut River Estuary." *The Wilson Bulletin* 104: 295–311.

Gnass Giese, E. E., R. W. Howe, A. T. Wolf, and G. J. Niemi. 2018. "Breeding Birds and Anurans of Dynamic Coastal Wetlands in Green Bay, Lake Michigan." *Journal of Great Lakes Research* 44(5): 950–959. https://doi.org/10.1016/j.jglr.2018.06.003.

Heath, S. R., E. H. Dunn, and D. J. Agro. 2020. "Black Tern (*Chlidonias Niger*), Version 1.0." In *Birds of the World*, edited by S. M. Billerman. Ithaca, NY: Cornell Lab of Ornithology. https://doi.org/10. 2173/bow.blkter.01.

Hickey, J. M., and R. A. Malecki. 1997. "Nest Site Selection of the Black Tern in Western New York." *Colonial Waterbirds* 20: 582. https://doi.org/10.2307/1521614.

Maxson, S. J., J. R. Fieberg, and M. R. Riggs. 2007. "Black Tern Nest Habitat Selection and Factors Affecting Nest Success in Northwestern Minnesota." *Waterbirds: The International Journal of Waterbird Biology* 30(1): 1–9. https://www.jstor.org/stable/4501788.

Prince, H. H., P. I. Padding, and R. W. Knapton. 1992. "Waterfowl Use of the Laurentian Great Lakes." *Journal of Great Lakes Research* 18(4): 673–699. https://doi.org/10.1016/S0380-1330(92)71329-X.

Wilcox, D. A. 2012. "Response of Wetland Vegetation to the Post-1986 Decrease in Lake St. Clair Water Levels: Seed-Bank Emergence and Beginnings of the Phragmites australis Invasion." *Journal of Great Lakes Research* 38(2): 270–277. https://doi.org/10.1016/j.jglr.2012.02.007.
