# Supplementary material for: Long‐term monitoring and remote sensing reveal waterbird colony collapses with rising lake levels and multiple stressors
Source: Ecol Appl. 2026 Sep 28;36(6):e70314. doi: 10.1002/eap.70314 (PMC13620196; doi:10.1002/eap.70314)
Supplement: Supplementary file 5 [file EAP-36-e70314-s005.pdf]

Appendix S3

Long-term monitoring and remote sensing reveal waterbird colony collapses with rising lake levels and multiple stressors

Jennifer L. Fuller, Erin Ford, Ava Landgraf, Johannes Foufopoulos, Andrew Gronewold, Karen M. Alofs

Ecological Applications

Nest Outcome

The following list enumerates (0-8) the possible nest outcomes and how researchers could make this determination. The outcomes were grouped into three survival categories to appropriately compare survival using a robust sample size (**Table S1**).

0 - Unknown egg survival - The nest was observed with adults incubating eggs on the last visit, but because it was not checked again, researchers could not determine whether the eggs hatched or failed. Alternatively, the nest was empty on the last visit, but it was unclear whether this was because the nest hatched or failed. This situation can often occur if the nest age and estimated hatch date are unknown, and the area was still active on the last visit (e.g., aggressive breeding adults are present and may be tending to a chick nearby).

1 - Egg fails - The nest was observed incubating previously, the last visit was made before it was close to its estimated hatch date, and no breeding adults are present in the area or clearly attending the nest (e.g., accurate flush counts provide clear evidence that all breeding adults are attending other local nests). If possible, the failed nest was recorded

as 1) “disappeared entirely” and could not be located on the last visit, e.g., the nest had completely broken apart or was washed away, but could not be confirmed as “destroyed by weather”, 2) “destroyed by weather” which was either confirmed with camera evidence or only assumed if the nest was washed away and experienced a 24-hour maximum lake level rise greater than 5 cm between visits (Clites et al., 2014), where camera footage confirmed that water level changes at 5 cm or above were capable of damaging nests, 3) “inact with missing eggs“ if the nest itself was present but both eggs and breeding adults were not, 4) “predated” if eggshells or punctered eggs were found, as adult terns typically remove eggshells after hatching (Cuthbert, 1954; and seen on nest cameras and observed in the field (**Figure S1**)), 5) “abandoned” if eggs were still present but breeding adults clearly no longer are attending the nest.

2 - Egg OR chick fails - The nest was observed incubating previously and found empty on the last visit, and no aggressive breeding adults were present and indicating they could be tending a chick nearby. Alternatively, accurate flush counts of the area provide clear evidence that all breeding adults present are actively tending to other nests. Researchers could not determine whether the eggs failed or the eggs hatched and subsequently failed because the age of the nest was unknown. The nest was also found empty <21 days after the previous visit (still incubating), which is before fledging would be possible and when adults and a fledgling could have left the area. A conservative threshold of 21 days is given because if the last observation during incubation was right before hatching, a chick would not be able to fledge until at least 21 days later.

3 - Chicks hatched, survival unknown - The nest was discovered with chicks at the nest. Sometimes nests could be deemed "hatched" with reasonable evidence without a chick spotted, if the nest had poop, feathers, and highly aggressive adults.

4 - Some chicks fail, rest are unknown – This is the case if a deceased chick was found at a nest.

5 - All chicks fail - The nest was rechecked after a known hatch, and no chicks or aggressive adults were present in the area. This is rarely a determinable outcome as chicks are capable of swimming away from the nest within days of hatching, and families are known to move their location over time. However, families are unlikely to move significant distances until chicks have fledged (21-24 days). Accurate flush counts of the area can provide evidence on whether the parents and chicks are still active in the sub-colony region (i.e., remaining adult pairs in the known area have incubating nests elsewhere).

6 - At least 1 chick fledged - A nest with monitored chicks had a successfully fledged chick which was spotted in the same area and fledged in the correct time period given the age of the chick/nest. Or, in another very rare case, an almost-fledged chick is located near an old nest and a fledgling is spotted in the same area within the same week – without any reason to believe it came from elsewhere. It is extremely rare to encounter this without frequent monitoring and nanotagging of chicks, which was carried out by Dr. Alex Jahn (2019). Even then, only 4 monitored nests from all years had confirmed fledglings given the almost impossible task of tracing their origin. Nest and chick ages and a firm understanding of the ages of nearby nests must be obtained in order to accurately determine where a spotted fledgling came from.

7 - At least 1 chick fledged and at least 1 sibling failed - A rare occurrence that was encountered only during frequent monitoring of chick survival and fledging by Dr. Alex Jahn (2019).

Table S1. Survival codes 0-8 simplified into egg survival with their associated sample size.

Survival Code	Original Codes	Count	Egg Survival
0	0, 2	370	unknown or unknown stage failed
1	1	165	eggs failed
2	3-7	286	eggs hatched

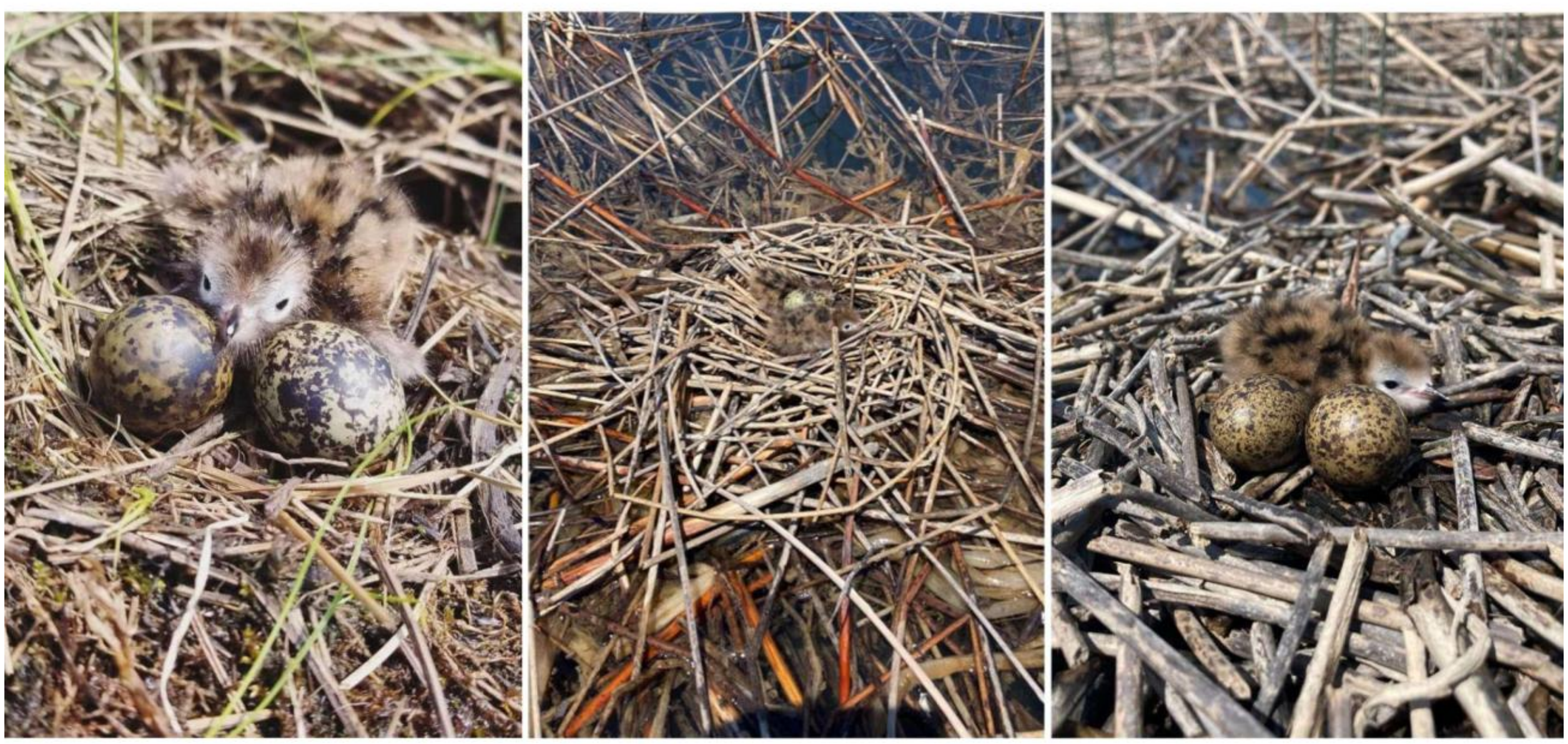

Figure S1. Photos of Black Tern nests at Wigwam Bay State Wildlife Area (right) and St. Clair Flats (middle, left) observed with a recently hatched chick(s) and eggshells removed (photo credits: Jennifer Fuller and Erin Ford).

Literature Cited

Clites, A. H., J. P. Smith, T. S. Hunter, and A. D. Gronewold. 2014. “Visualizing Relationships between Hydrology, Climate, and Water Level Fluctuations on Earth’s Largest System of Lakes.” *Journal of Great Lakes Research* 40(3): 807–811. https://doi.org/10.1016/j.jglr.2014.05.014.

Cuthbert, N. L. 1954. “A Nesting Study of the Black Tern in Michigan.” *The Auk* 71(1) 36-63. https://doi.org/10.2307/4081515.
