# Supplementary material for: Long‐term monitoring and remote sensing reveal waterbird colony collapses with rising lake levels and multiple stressors
Source: Ecol Appl. 2026 Sep 28;36(6):e70314. doi: 10.1002/eap.70314 (PMC13620196; doi:10.1002/eap.70314)
Supplement: Supplementary file 6 [file EAP-36-e70314-s006.pdf]

Appendix S2

Long-term monitoring and remote sensing reveal waterbird colony collapses with rising lake levels and multiple stressors

Jennifer L. Fuller, Erin Ford, Ava Landgraf, Johannes Foufopoulos, Andrew Gronewold, Karen M. Alofs

Ecological Applications

Estimating Nesting Time Frame

Given Black Tern re-nesting ability, colonial nature, and high chick mobility, identifying nest survival required careful observations by field researchers and an understanding of the nest's age when possible. Identifying the starting date of each nest also provides important temporal information, especially for identifying seasonal initiation and what changing environmental variables these nests experience (i.e., wind and lake fluctuation). Although some nests were found by chance at a very early stage (i.e., 0 eggs, about to begin laying), most nests were found later during incubation or when they had already hatched. To determine the starting date (whenever possible) we used the following criteria and the summary table listed in **Table S1**.

1. Egg Laying Stage (2-3 days)

Egg-laying is typically carried out at 1-day intervals, with eggs at this early stage sinking fully when placed in water (*1*).

2. Incubation Stage (21-22 days)

Once the typical 3-egg clutch is completed and/or eggs are no longer fully sinking, incubation begins and nest age estimates are adapted from Hays and LeCroy, 1971 (Hays & Lecroy, 1971). These floating stages were determined using a proxy species (Common Tern, *Sterna hirundo*); however, this method is also accurate for Black Terns and has been used in other Black Tern studies including (Zuben & Nocera, 2019). Length of the exposed shell above water was also adapted to the smaller width of Black Tern eggs (21.9-26.1 mm) compared to the common tern (31-34 mm) (Heath et al., 2020) by multiplying the exposed diameter of the common tern with the ratio (0.738) of average Common Tern (32.5 mm) and Black Tern (24 mm) diameter (**Table S2**).

3. Chick (Pre-Fledge) Stage (21-24 days)

When chicks are found, they are typically either assessed visually for age (typically between 0-3 days old) by the researcher or banded and weighed when they can be safely caught. Chick weight can be used to estimate both bird age and age of the nest, and there are multiple methods that can be used. Regardless of the method, if multiple chicks are weighed from the same nest, the largest chick is used to estimate the nest's age (or the oldest, if a larger chick was measured on a later date from the same nest) as it will estimate the earliest start date. The growth rate of chicks under 10 days of age has been identified in multiple sources (Bailey, 1977 p. 19; Einsweiler, 1988). Their results indicate a weight gain of 4.18-5.18 grams/day. However, this average growth rate is not linear and varies, depending on their age.

Using the weight of chicks measured at St. Clair Flats (Alex Jahn, pers. comm.), a polynomial growth curve was first produced to more finely estimate chick age and attempt to consider potential temporal changes during development. The slope was first taken from two chicks that were weighed upon hatching and 1 or 2 times following ($y = 5.9419x + 8.5508$, R Square = 0.8112). This slope was applied to chicks measured 2-3 times for weight to estimate their estimated hatch date (n=10). Their consecutive weights were then plotted against the date and time they were measured. If the time of day was not recorded, the average hour of 11:33 am for chick weighing was applied. A polynomial curve was applied to a scatterplot, which had a greater R-squared than a linear regression (0.947) and accounted for the maximum chick weight in the 60-70 g range. Both the polynomial curve and literature suggest that chick growth is largely sustained within the first 10 days (Bailey, 1977; Einsweiler, 1988). Therefore, age was not measured once the chick was over 50 grams (under 10 days old). However, a nest was still aged if a smaller sibling was located, in which case 1 day was added to the nest's total age assuming the chicks hatched within consecutive days of each other. The polynomial curve and equation are shown in **Figure S1**.

Given the fact that 10 days was the cutoff for accurate age estimation, a simpler, derived linear regression was applied using only weights under 10 days (n = 6) (**Figure S2**). This estimation was slightly higher (5.335 g/day) in comparison to the previously cited studies (4.18-5.18 g/day). If the chicks had fledged, there were some rare circumstances where this information could determine the age of the nest. The observations had to be able to identify "before" and "after" fledging dates and were acquired from a known nest. Chicks

are known to fledge between 21 and 24 days of age (Heath et al., 2020). Once chick or fledgling age was successfully estimated, the age of the nest was then calculated by simply subtracting 23 and 24 days (late and early estimates). A full table summary of egg estimation methods can be viewed in **Table S2**.

If multiple estimates were collected, we chose the most accurate method using the following criteria. The most accurate measurement was when the nest was found or observed on camera at the earliest stage (e.g., 1 or 2 eggs, sinking), located again with 1 or 2 more eggs, and had clearly not re-nested in that time. In this case, the nest was found within the first two days of incubation and had 0 days of error **(Table S1)**. The second most accurate method was to back-calculate from when a chick was found clearly in the hatching process, either using camera evidence or if the egg was found either pipped or hatching. Calculating chick age using their weight was considered the third most accurate measurement up to 10 days of age, where their weight plateaus **(Figure S1 and S2)**. Float tests between stages a through i **(Table S1)** were the fourth most accurate method, given that floating eggs does not use precise measurements such as weight, and leaves more room for subjectivity. Finally, using fledgling age was considered the least accurate estimate **(Table S1)** and was only used in rare circumstances when the observations included "before" and "after" fledging dates, and could be traced to its nest of origin.

Table S1. Egg age estimation algorithm, adapted from Hays and Lecroy (1971).

Letter	Description	Age Estimate (Days) Young	Midpoint	Old	Error
a0	0 eggs, 1 day prior to laying	0	0	0	0
a1	1 fresh, full sink	1	1	1	0
a2	2 fresh, full sink	2	2	2	0
a	3 fresh, full sink	3	3.5	4	1
b	full sink, slight lift	5	5.5	6	1
c	sink, tilting but not upright	7	7.5	8	1
d	sinking vertically	9	9.5	10	1
e	float, not breaking the surface or 11 mm exposed shell	11	11.5	12	1
f	float, breaking surface (13 mm)	13	13.5	14	1
g	float, breaking surface (15 mm)	15	15.5	16	1
h	float, breaking surface (16 mm)	17	17.5	18	1
i	float, breaking surface (18 mm)	19	19.5	20	1
j	float, breaking surface (18 mm), shell pipped	21	21.5	22	1
k	in process of hatching	23	23.5	24	1
fl	fledged	21	22.5	24	3
	chick growth to fledging + egg incubation + egg laying	44	46	48	4
hatch	chick found and weighed or estimated age visually	age depends on field estimate			varies
	chick age + incubation	add 23	add 23.5	add 24	1

Table S2. Exposed shell dimensions applied to nest age survey calculation.

**Type**	**Letter Stage**	**Common Tern**	**Black Tern**
Average width (mm)	NA	32.5	24
	e	15	11
	f	17	13
Exposed Shell Diameter (mm)	g	20	15
	h	21	16
	i	25	18

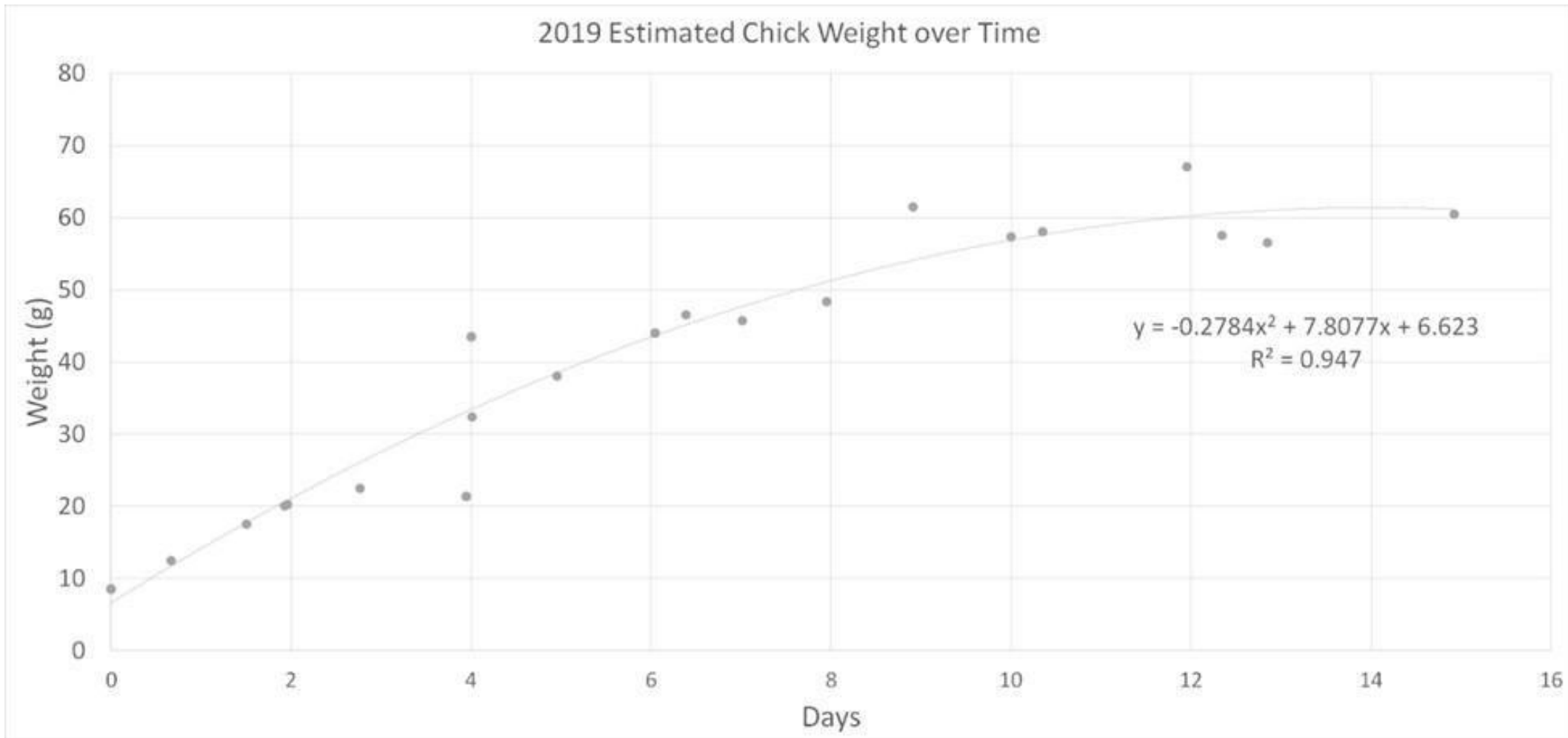

Figure S1. Polynomial curve of estimated chick weight against their age (days) using weights (g) measured 2-3 times per chick; n = 10.

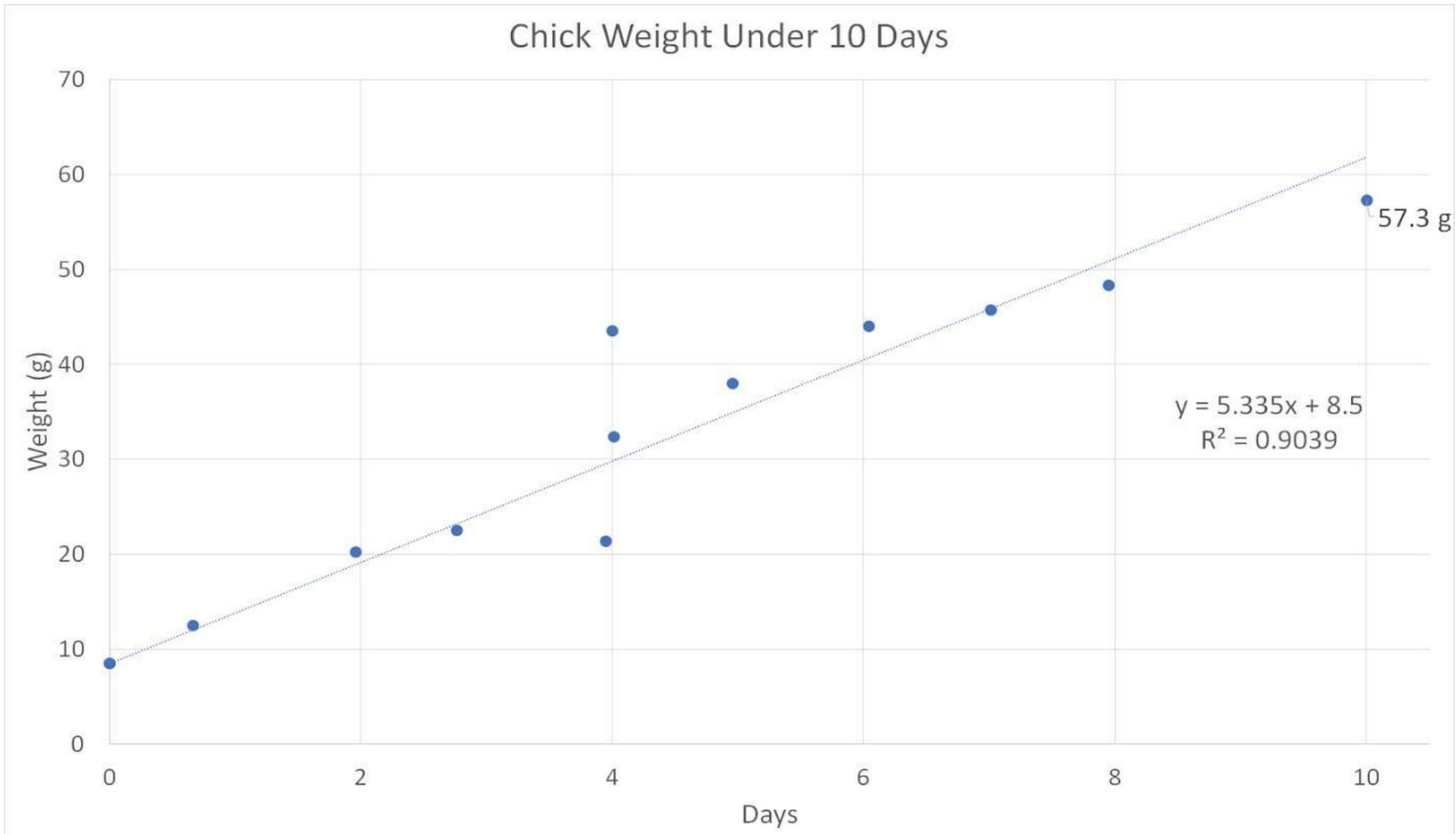

Figure S2. Simplified linear growth of chick against their age (days) using weights (g) measured 23 times per chick, under 10 days old; n = 6.

Literature Cited

Bailey, P. F. 1977. *The Breeding Biology of the Black Tern (Chlidonias niger surinamensis Gmelin).* Oshkosh, WI: University of Wisconsin Oshkosh.

Einsweiler, S. S. 1988. *Black Tern Nesting Biology in Cheboygan County, Michigan.* Mount Pleasant, MI: Central Michigan University.

Hays, H., and M. Lecroy. 1971. "Field Criteria for Determining Incubation Stage in Eggs of the Common Tern." *The Wilson Bulletin* 83(4): 425–429.

Heath, S. R., E. H. Dunn, and D. J. Agro. 2020. "Black Tern (*Chlidonias Niger*), Version 1.0." In *Birds of the World*, edited by S. M. Billerman. Ithaca, NY: Cornell Lab of Ornithology. https://doi.org/10. 2173/bow.blkter.01.

Zuben, V. V., and J. J. Nocera. 2019. "Drivers of Intra-Seasonal Variation in Black Tern (*Chlidonias niger*) Nest Survival." *Waterbirds* 42(1): 112–121.
