# Supplementary material for: Long‐term monitoring and remote sensing reveal waterbird colony collapses with rising lake levels and multiple stressors
Source: Ecol Appl. 2026 Sep 28;36(6):e70314. doi: 10.1002/eap.70314 (PMC13620196; doi:10.1002/eap.70314)
Supplement: Supplementary file 7 [file EAP-36-e70314-s007.pdf]

Appendix S9

Long-term monitoring and remote sensing reveal waterbird colony collapses with rising lake levels and multiple stressors

Jennifer L. Fuller, Erin Ford, Ava Landgraf, Johannes Foufopoulos, Andrew Gronewold, Karen M. Alofs

Ecological Applications

Post-hoc Evaluation of 2017 Classified Habitat Composition

To better interpret the composition of the dense habitat class, we conducted a post-hoc comparison using a 2017 Landsat and PALSAR-classified wetland habitat dataset of the region (Bourgeau-Chavez et al., 2019). Percentages of Landsat and PALSAR-classified landcover types were extracted using the “tabulate intersection tool” in ArcGIS Pro within each of the corresponding habitat types classified in 2017 for this study (sparse vegetation, medium, dense vegetation, island developments, open water).

Table S1. Percentages of Landsat and PALSAR-classified landcover (Bourgeau-Chavez et al., 2019) within each of the corresponding habitat types classified in 2017 for this study (sparse, medium, and dense vegetation, island developments, and open water).

Classified Habitat	Non-wetland landcover[1]	Non-emergent aquatic and wetland landcover[2]	Schoenoplectus	Typha	Phragmites	Water
Sparse Vegetation	1.71	2.65	61.52	0.40	1.53	32.19
Medium Vegetation	1.22	2.67	56.91	0.90	10.98	27.32
Dense Vegetation	2.53	2.20	25.42	1.31	45.40	23.14
Island Developments	49.63	1.27	28.37	1.79	15.12	3.82
Open Water	0.42	0.35	11.63	0.04	0.19	87.37

[1]Includes suburban, grass, paved surfaces, agriculture, forest, forested wetland, shrub wetland, and barren

[2]Includes forested wetland, forested shrub, aquatic bed, wet meadow

Literature Cited:

Bourgeau-Chavez, L. L., K. P. Kowalski, M. J. Battaglia, and A. F. Poley. 2019. "Land cover map including wetlands and invasive Phragmites circa 2017." U.S. Geological Survey data release: https://doi.org/10.5066/P9OX21T6.
