# Supplementary material for: Long‐term monitoring and remote sensing reveal waterbird colony collapses with rising lake levels and multiple stressors
Source: Ecol Appl. 2026 Sep 28;36(6):e70314. doi: 10.1002/eap.70314 (PMC13620196; doi:10.1002/eap.70314)
Supplement: Supplementary file 8 [file EAP-36-e70314-s008.pdf]

Appendix S6

Long-term monitoring and remote sensing reveal waterbird colony collapses with rising lake levels and multiple stressors

Jennifer L. Fuller, Erin Ford, Ava Landgraf, Johannes Foufopoulos, Andrew Gronewold, Karen M. Alofs

Ecological Applications

Imagery Preprocessing and Metadata

4-band, 1 m and 5 m spatial resolution images were collected from multiple sources for geospatial habitat measurements. Images with 1 m spatial resolution were used to capture fine-scale vegetation and open water classes. Sources included commercial satellite images (Kompsat-2, Triplesat-3, WorldView-2 and 3), and open-source aerial photographs from the National Agriculture Imagery Program (NAIP). All images expect Kompsat-2 were obtained geometrically preprocessed. Kompsat-2 was geometrically corrected at 1:5000 to 1 m resolution (1st Order Polynomial), geometrically corrected NAIP 2016 image. 2013, 2017, and 2019 images were pansharpened using the Brovey method in ArcMap. Kompsat-2, Worldview-2, and Worldview-3 image bands were individually converted to at-sensor radiance (W m-2 sr-1 µm) and top of atmosphere reflectance (TOA). Dark object subtraction was then performed to control for reflectance variations between different times. Methodology followed procedures, equations, sensor-specific calibration coefficients, and provided metadata detailed in their associated literature (**Table S3)**.

Kompsat-2 used the following equation and gain/offset values from Lee et al. 2011 (Lee et al. 2012):

$$(1a)\ L\lambda = GAIN \times DN + OFFSET$$

WorldView-2 and WorldView-3 used the same equation described in Comp & Updike 2011, using updated gain and offset values from Kuester, 2017 (Kuester 2016). Abscalfactor and effectivebandwith are provided for each band in the imagery metadata:

$$(1b)\ L\lambda = GAIN \text{ x } DN \text{ x } (abscalfactor/effectivebandwith) + OFFSET$$

To convert to TOA reflectance, all sensors utilized the following relationship:

$$(2)\ p\lambda = \pi \times L\lambda \times d2 / ESUN\lambda \times \cos \theta SZ$$

pλ is band λ TOA reflectance, d is earth-sun distance (astronomical units). ESUNλ is the average band solar exoatmospheric irradiance (W/m2 /µm), values provided for Rapideye-5, Kompsat-2, and WorldView-2/3 using the following references, respectively (Lee et al. 2012, Kuester 2016, Hojas Gascón et al. 2019). θSZ is the solar zenith angle calculated by subtracting the solar elevation angle provided in the metadata from 90°.

NAIP imagery was downloaded geometrically and radiometrically corrected. Atmospheric effects were corrected using dark object subtraction based on histograms of each band as described in Chavez (1988). The Triplesat-3 image was received already radiometrically and atmospherically corrected as a surface reflectance (SR) product (**Table S3**).

Unfortunately, images under 1-m spatial resolution were limited in temporal availability, particularly when the focal season and habitat changes are occurring within a span of a

few months. Therefore, mean NDVI was also used as a more temporally holistic, coarser scale (5 m) proxy of emergent vegetation density. Images for NDVI were collected from Planet as geometrically and atmospherically preprocessed as surface reflectance (SR) products (Rapideye-5 and PlanetScope).

NDVI and NDWI calculations were performed in ArcMap using the built-in Image Analysis function and Raster Calculator, consecutively, which utilize the following equations:

$$(1)\ NDVI = (NIR - RED) / (NIR + RED)$$

$$(2)\ NDWI = (GREEN - NIR) / (GREEN + NIR)$$

Commercial and NAIP NDVI and NDWI raster images were then resampled to 1 m resolution; and Rapideye-5 and PlanetScope to 5 m resolution using bilinear interpolation in ArcMap.

Ideally, all images used in NDVI and NDWI analysis would be collected on the same anniversary date each year. Unfortunately, however this is not yet feasible with high-spatial resolution images. For high spatial resolution imagery, the best compromise was to utilize images as close in timing during the breeding season as possible, and after the advent of "leaf-on" to best differentiate classes. All high spatial resolution images were collected within 9 days of each other (standard deviation = 8.71 days) between late June and July (**Table S2**). In 2016, visual interpretation and classification of both available images (6/22/2016 and 8/6/2016 dates) showed strong under or overestimation of NDVI values due to the early and late stages in the seasonal growth period. The NDVI raster

from 2016 was linearly interpolated between the image from 6/22/2016 and 8/6/2016 to an estimated date of 7/16/2016. This unfortunately assumes a linear relationship in NDVI over time, which is unlikely to be wholly accurate. However, this reduced the standard deviation of dates among years and prevented over or under class estimation bias, which was more desirable than choosing one or the other.

As the temporal resolution was somewhat improved (increasing each year) using PlanetScope and Rapideye-5 (5-m), their NDVI and NDWI raster images were linearly interpolated between the closest two dates to the latest possible date (prior to vegetation senescence) during the nesting season (July 11th) to create anniversary images for comparison (**Table S1**).

Table S1. Collection summary table of Planet Images (Rapideye-5 & PlanetScope) used for mean NDVI collection including dates, error, original spatial resolution, and source. A stand-in NDVI raster was generated by linearly interpolating between the earliest and latest image. “Error (Day Span)” is the number of days between the two images used to create the interpolated image.

Planet Imagery (5-m) Temporal and Spatial Resolutions					
**Year**	**Earliest Image**	**Latest Image**	**Error (Day Span)**	**Spatial Resolution (m)**	**Source**
2013	5/16/2012	7/13/2013	58	5	Rapideye-5
2014	6/21/2014	8/17/2014	57		
2015	7/6/2016	7/23/2015	17		
2016	7/8/2016	7/26/2016	18		
2017	7/4/2017	7/23/2017	19	3	PlanetScope
2018	7/8/2018	7/25/2018	17		
2019	7/11/2019	7/11/2019	0		
2020	7/4/2020	7/25/2020	21		

Table S2. Collection summary table of images with a final pansharpened spatial resolution <1-m (KS-2, NAIP, TS-3, and WV-2) used for fine-scale habitat classification and distances. Includes collection dates, multi-spectral spatial resolution, panchromatic spatial resolution, and source by year

High Resolution (1-m) Temporal and Spatial Resolutions				
**Year**	**Date(s)**	**MS Spatial Resolution (m)**	**Panchromatic Spatial Resolution (m)**	**Source**
2013	7/13/2013	5	1.1	Kompsat-2
2014	7/5/2014	1	NA	NAIP
2015	NA	NA	NA	NA
2016	6/22/2016<br>8/6/2016	2.8	0.7	TripleSat-3<br>NAIP
2017	7/31/2017	2.8	0.7	WorldView-2
2018	7/18/2018	0.6	NA	NAIP
2019	7/20/2019	2	0.5	WorldView-2
2020	7/7/2020	2	0.5	WorldView-2

Table S3. Pre-processing summary table of images with a final pansharpened spatial resolution <1 m (KS-2, NAIP, TS-3, and WV-2) used for fine-scale habitat classification and distances. Includes collection dates, source, atmospheric and normalization correction types, additional processing types, and whether interpolation was required by year. Atmospheric and normalization techniques include Top of Atmosphere (TOA), Dark Object Subtraction (DOS), and Surface Reflectance (SR). Additional processing includes Pan Sharpening, Brovey Method (PS), and georectification.

**High Resolution (1-m) Corrections, Processing, and Interpolation**					
**Year**	**Date(s)**	**Source**	**Atmospheric & Normalization Correction**	**Additional Processing**	**Interpolation**
2013	7/13/2013	Kompsat-2	TOA + DOS	PS + Georectification	N
2014	7/5/2014	NAIP	DOS	None	N
2016	6/22/2016	TripleSat-3	SR + DOS	PS	Y
	8/6/2016	NAIP	DOS	None	
2017	7/31/2017	WorldView-2	TOA + DOS	PS	N
2018	7/18/2018	NAIP	DOS	None	N
2019	7/20/2019	WorldView-2	TOA + DOS	PS	N
2020	7/7/2020	WorldView-2	TOA + DOS	PS	N

Literature Cited

Chavez, J., Pat. 1988. "An Improved Dark-Object Subtraction Technique for Atmospheric Scattering Correction of Multispectral Data." *Remote Sensing of Environment* 24(3): 459–479.

Hojas Gascón, L., G. Ceccherini, F. J. García Haro, V. Avitabile, and H. Eva. 2019. "The Potential of High Resolution (5 m) RapidEye Optical Data to Estimate Above Ground Biomass at the National Level over Tanzania." *Forests* 10(2): 107.

Kuester, M. A. 2016. "Absolute Radiometric Calibration: 2016v0 GE01 and CAVIS." Digital Globe.

Lee, S., C. Jin, C. Choi, H. Lim, Y. Kim, and J. Kim. 2012. "Absolute Radiometric Calibration of the KOMPSAT-2 Multispectral Camera Using a Reflectance-Based Method and Empirical Comparison with IKONOS and QuickBird Images." *Journal of Applied Remote Sensing* 6(1): 3594.
