# Supplementary material for: Long‐term monitoring and remote sensing reveal waterbird colony collapses with rising lake levels and multiple stressors
Source: Ecol Appl. 2026 Sep 28;36(6):e70314. doi: 10.1002/eap.70314 (PMC13620196; doi:10.1002/eap.70314)
Supplement: Supplementary file 9 [file EAP-36-e70314-s009.pdf]

Appendix S4

Long-term monitoring and remote sensing reveal waterbird colony collapses with rising lake levels and multiple stressors

Jennifer L. Fuller, Erin Ford, Ava Landgraf, Johannes Foufopoulos, Andrew Gronewold, Karen M. Alofs

Ecological Applications

Water Depth Data Collection

The depths of the lake below individual nests were infrequently collected in the field, with only samples taken in 2018, 2019, and 2020 and even then, only to varying amounts. In many cases, getting close enough to the nest to take this measurement would disturb the nest too much and can cause destruction of the surrounding mat, especially in later years when the lake level was high. However, as an alternative we used the NOAA Lake Level Viewer Tool. This offers an open-source download of a high-resolution (3 m) DEM created by the NOAA Office for Coastal Management using topobathy lidar and dredge survey data (USACE), and multibeam sonar (NPS) (Roth et al., 2016). The "sample" function in ArcMap was used to derive elevation (nearest neighbor) for as many nests as possible from the NOAA DEM and nest GPS locations.

The HECWFS dataset (Clites et al., 2014) was used to derive the water depth (cm) below the nest by subtracting the DEM values from the average HECWFS surface lake level across the nesting (St. Clair Flats) region. HECWFS datapoints were chosen by using the

“generate near table” function in ArcMap, collecting the 4 nearest water level points to all nests (**Figure S1**). Wind data points were also included during this step in data collection (2 nearest wind points) to identify whether storms significantly contributed to nest failure. However, the data resolution was too low to include in the final analysis. All the resulting nest GPS locations had associated HECWFS data from each year, regardless of whether the nest occurred. This ensured that the overall weather data was representative of the full nesting region. Raw data was converted from their native, separate txt files by point and year to combined excel files by year. Lake level data remained in its original format as surface elevation (m). The temporal resolution of the HECWFS model was 3 hours, which was important for the initial measure of whether storms contributed to failures. Lake level data was assigned to sub-colony regions with some overlap and were compared within R for any major differences in fluctuation. Outliers were then removed from each sub-colony, and an average was taken for each sub-colony region. This simplified the data to an appropriate level.

For all nests, the depth date was standardized to the onset of the season (5/15). The use of HECWFS and nest DEM was also compared to field depth measurements. This was performed by taking the average 24-hr lake level from HECWFS or Algonac on the same day the field depth (n = 102) was taken and plotting against one another. Unfortunately, in comparison to field measurements, the R^2 value was low (0.080). However, this is not entirely surprising since the water depth can vary widely in regions immediately surrounding each nest. In this case, bathymetric elevation likely represents the depth in a

surrounding 9 m^2 of the nest and becomes a metric of the surrounding habitat rather than a singular point of reference at the nest itself.

Given these uncertainties regarding the true water depth values directly below the nest, and to improve interpretability, water depth values were converted to relative change in cm with the shallowest value set at 0.

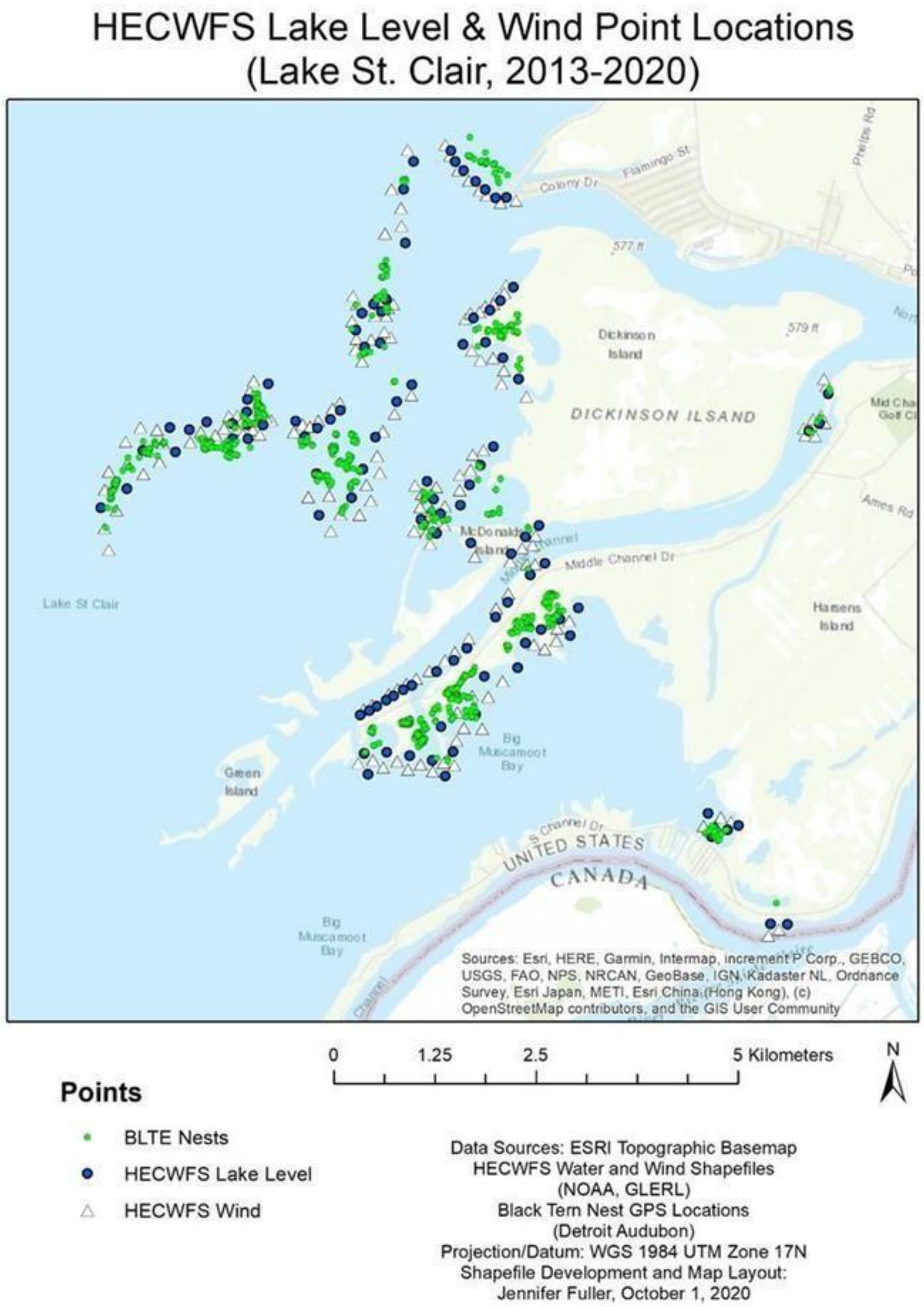

Figure S1. Point locations of nests from all years (2013-2020) in the SCF region, with associated wind and lake level locations where HECWFS 3-hour values are derived. Lake level locations are the same locations in which air temperature values are also collected.

Literature Cited

Clites, A. H., J. P. Smith, T. S. Hunter, and A. D. Gronewold. 2014. "Visualizing Relationships between Hydrology, Climate, and Water Level Fluctuations on Earth's Largest System of Lakes." *Journal of Great Lakes Research* 40(3): 807–811. https://doi.org/10.1016/j.jglr.2014.05.014.

Roth, R., D. Hart, R. Mead, and C. Quinn. 2016. "Wireframing for Interactive & Web-Based Geographic Visualization: Designing the NOAA Lake Level Viewer." *Cartography and Geographic Information Science* 44(4): 338–357. https://doi.org/10.1080/15230406.2016.1171166.
