# Supplementary material for: Long‐term monitoring and remote sensing reveal waterbird colony collapses with rising lake levels and multiple stressors
Source: Ecol Appl. 2026 Sep 28;36(6):e70314. doi: 10.1002/eap.70314 (PMC13620196; doi:10.1002/eap.70314)
Supplement: Supplementary file 10 [file EAP-36-e70314-s010.pdf]

Appendix S10

Long-term monitoring and remote sensing reveal waterbird colony collapses with rising lake levels and multiple stressors

Jennifer L. Fuller, Erin Ford, Ava Landgraf, Johannes Foufopoulos, Andrew Gronewold, Karen M. Alofs

Ecological Applications

Spatial Predictor Selection of Hatching Success GLMM

Table S1. Summary of generalized linear mixed-effects model (GLMM) selection results predicting hatching success to determine the appropriate spatial scale of % dense vegetation and NDVI predictors. Six candidate model sets were created using combinations of % dense vegetation (3, 7, or 12-m radius) and mean NDVI (7 or 12-m radius). For each combination, candidate models were created using dredge() from global models containing a consistent set of core predictors (number of nests within a 30-m radius, clutch initiation, relative initial nest water depth, house distance, lake distance, and open water distance) with sub-colony included as a random intercept. The table reports the best-supported model output from each model input combination, ranked by Akaike's Information Criterion (AIC). Δ AIC values are calculated relative to the top-ranked model across all model combinations, and model performance is summarized as the receiver operating characteristic curve (AUC).

Dense Vegetation radius (m)	NDVI radius (m)	Best-supported predictors	AIC	ΔAIC	AUC
12	7	Open water distance + % Dense vegetation (12 m) + Mean NDVI (7 m)	397.67	0	0.8429
12	12	Open water distance + % Dense vegetation (12 m)	398.98	1.31	0.8418
3	7	% Dense vegetation (3 m) + Mean NDVI (7 m)	402.55	4.88	0.8302
3	12	% Dense vegetation (3 m)	403.37	5.7	0.8299
7	7	Open water distance + % Dense vegetation (7 m) + Mean NDVI (7 m)	406.02	8.35	0.8301
7	12	Open water distance + % Dense vegetation (7 m)	407	9.33	0.8287

*All top models produced by dredge() contained clutch initiation, relative initial nest water depth, and lake distance
